# Supplementary material for: Spontaneous emergence of skyrmions beyond room temperature in boron-doped CrTe
Source: Commun Mater. 2026 Aug 13;7(1):210. doi: 10.1038/s43246-026-01336-1 (PMC13472906; doi:10.1038/s43246-026-01336-1)
Supplement: Supplementary file 1 [file 43246_2026_1336_MOESM1_ESM.pdf]

Supplementary information for

Spontaneous emergence of skyrmions beyond room temperature in boron doped CrTe

Jagannath Jena[1,4,#,*], Konstantin Holst[1,2,#], Börge Göbel[2,#], Yangkun He[3], Binoy Krishna Hazra[1], Pedram Bassirian[1], Katayoon Mohseni[1], Georg Woltersdorf[2], Ingrid Mertig[2], Holger Meyerheim[1], Claudia Felser[3], and Stuart S. P. Parkin[1*].

[1]Max Planck Institute of Microstructure Physics, Halle (Saale), Germany.

[2]Institute of Physics, Martin Luther University Halle-Wittenberg, Halle (Saale), Germany.

[3]Max Planck Institute for Chemical Physics of Solids, Dresden, Germany

[4]Materials Science Division, Argonne National Laboratory, Illinois, USA

#These authors contributed equally.

*Corresponding authors: jjena@anl.gov

stuart.parkin@mpi-halle.mpg.de

Supplementary Information

1. X-ray analysis of the $Cr_{0.9}B_{0.1}Te$ crystal structure

The structural analysis of the $Cr_{0.9}B_{0.1}Te$ single crystal was carried out using a six-circle diffractometer equipped with a two-dimensional pixel detector and a Ga-jet x-ray source (λ=1.341 Å) operated at 70 kV and 200 W power[1]. A Montel-optics focuses the monochromatic beam on the sample with a beam size of 100µm × 2 mm. The structural refinement was carried out by least squares refinement of the calculated squared structure intensities, $|F_{hkl}|^2$ to the experimental ones. The latter were derived from the integrated reflections intensities collected by transverse phi-scans about the normal of the (0001) surface of the sample.

Supplementary Figure 1 shows a schematic of the structural model derived from the refinement. One unit cell is shown, where green and red balls represent Cr and Te atoms, respectively. Boron atoms were not considered as their scattering power is negligible. First of all, the structure of our CrTe crystal cannot be mapped to the space group (SGR) $P6_3/mmc$, since we clearly observe reflections with scattering vectors $\overrightarrow{(Q)}$ of type $\vec{Q}$= (H, H, $\overline{2H}$, L) with L being an odd number, which are forbidden in SGR #194. This observation provides direct evidence for a lower symmetry than $P6_3/mmc$ as reported in the past for PtMnGa single crystal and the group of Fe_xGeTe_2 crystals[2, 3]. Artifacts such as the contribution of the allowed second order reflection $2\vec{Q}$ excited by λ/2 can be ruled out by the strong suppression of the higher harmonic by the Montel optics.

The consideration of subgroups to $P6_3/mmc$ which allow reflections of type $\vec{Q}$= (H, H, $\overline{2H}$, L) only leaves SGR #156 (P3m1) and SGR #164 ($P\bar{3}2/m$) as suitable choices. Our detailed analysis based on 21 symmetry independent reflections suggests that the structure of our crystals belongs to SGR #164, which is centrosymmetric. Suppl. Table1 lists the atomic coordinates and disorder parameters (U) of all symmetric independent atoms with in the unit cell. Allowing for further symmetry reduction to the acentric SGR #156 (P3m1) led to a fit quality which is several percent worse. In this analysis the fit quality is characterized by the weighted residual (wR2) and the goodness of fit (GOF) parameter[4, 5]. In detail we find for SGR #164, wR2=0.228 and GOF=1.186, while for SGR #156 wR2=0.229 and GOF=1.239 was found, the latter being more significant as GOF takes into account the difference between the number of refined parameters and the number of reflections. Albeit the difference is small we conclude in combination with the results of the electron diffraction analysis that the CrTe

crystal is characterized by the centrosymmetric SGR 164. In addition, we found some evidence that about 10% of Cr occupies site 2d at (x, y, z)=($^{2}/_{3}$, $1/_{3}$, 0.350).

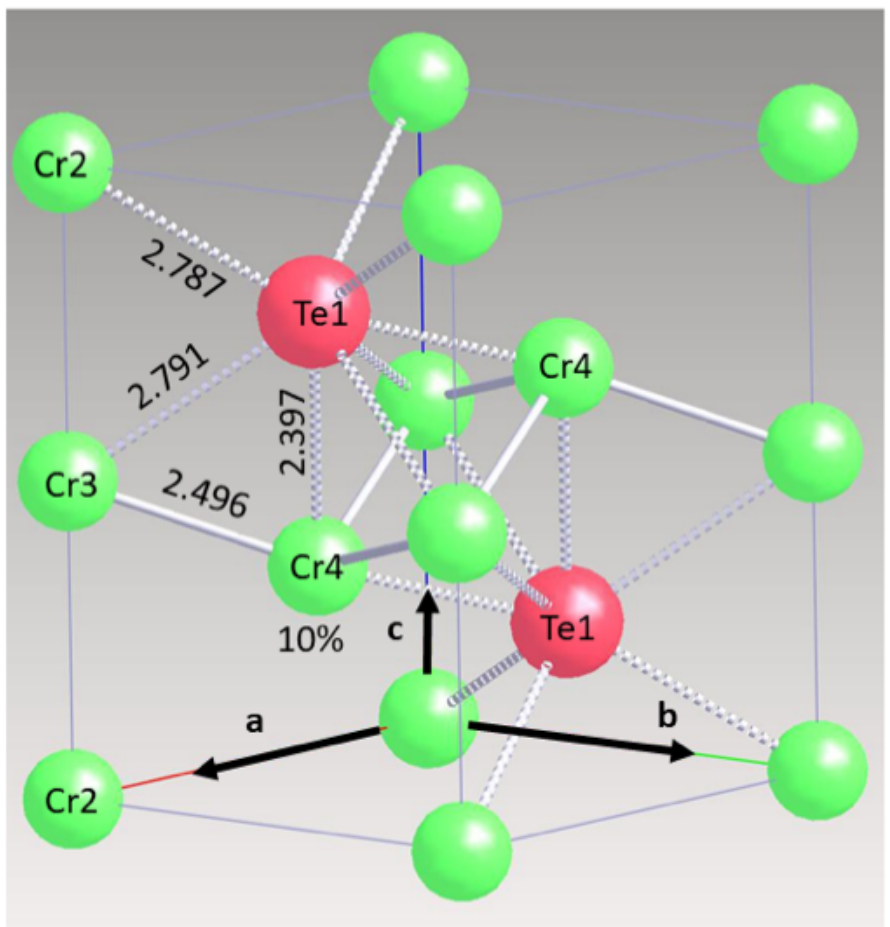

Suppl. Fig. 1: X-ray structural analysis of the CrTe single crystal: Schematic of the CrTe crystal structure. One unit cell is shown in perspective view. Green and red balls represent Cr and Te atoms, respectively. Atoms are labelled according to Suppl. Table 1. Note, that the site Cr4 is only partially occupied by about 10%. Interatomic distances are given in Ångstrøm units. Thermal ellipsoids are not shown.

Atom<br><br>(Wyckoff site)	Θ	x	y	z	$U^{11}=U^{22}$, U^{33} (Å^2)
Te1 (2d)	1.00	1/3*	2/3*	0.249±001	0.005±0.004, 0.090±0.001
Cr2 (1a)	1.00	0.0*	0.0*	0.0*	0.016±0.009, 0.077±0.010
Cr3 (1b)	1.00	0.0*	0.0*	0.5*	0.016±0.009, 0.077±0.010
Cr4 (2d)	0.10	2/3*	1/3*	0.350±0.007	0.016±0.009, 0.077±0.010

Suppl. Table 1: Coordinates of the atoms in the CrTe structure: Refined positions in relative coordinates within the unit cell and Debye parameters (B) of the atoms in space group #164 (P$\bar{3}$2/m). Lattice parameters are a = b = 4.008 Å, c = 6.229 Å. Labels refer to Supplementary Figure 1, labels in parentheses refer to the Wyckoff position. Parameters marked with asterisks were kept fixed. Atomic displacement parameters for Cr atoms were set to be equal. Note the strong out of plane enhancement of the atomic displacement parameters ($U^{33} > U^{11}$) observed for all atoms.

Supplementary Figure 2

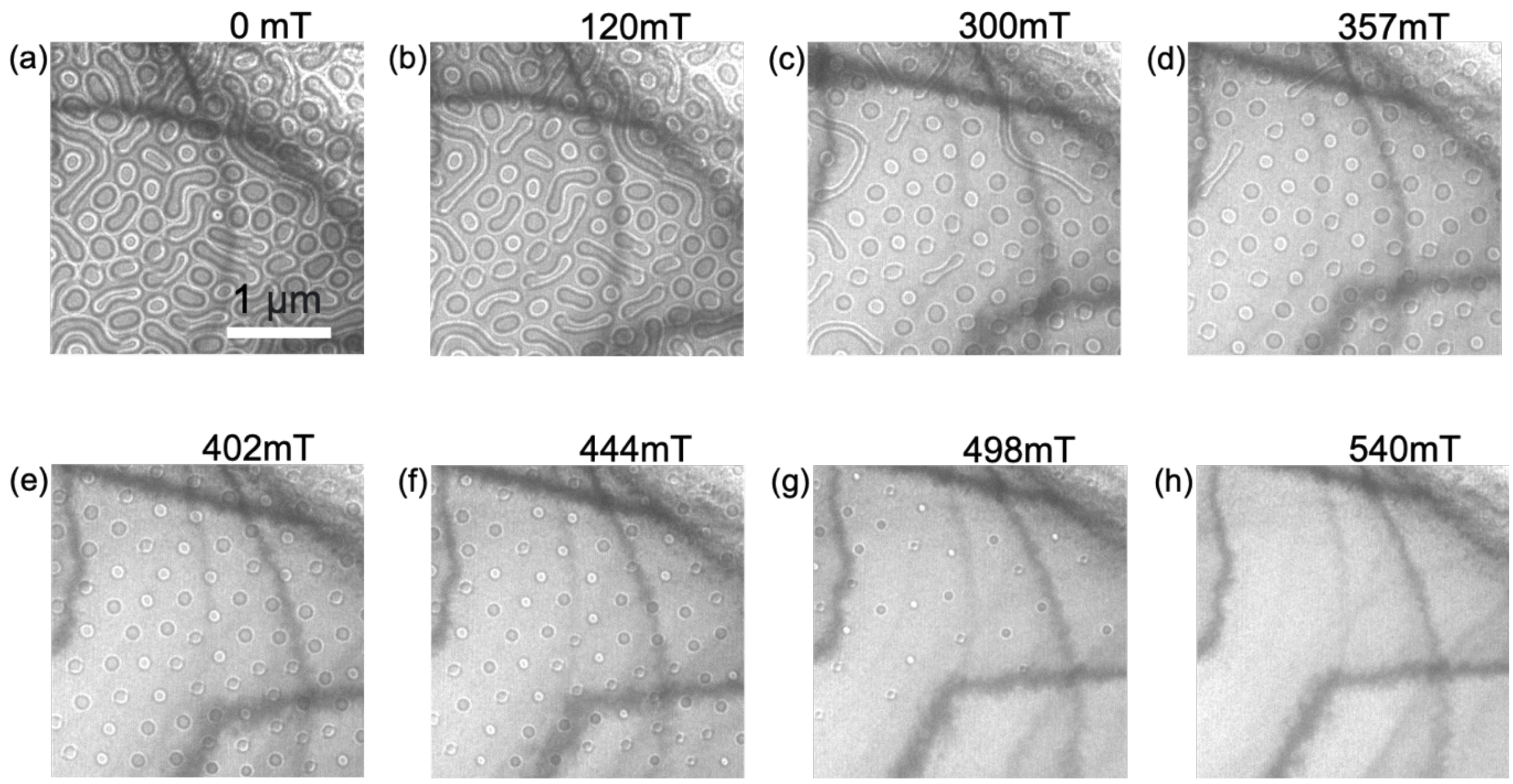

Suppl. Fig. 2: Field evolution of spontaneous spin textures at 100 K. (**a**) Zero field spontaneous skyrmions and short spiral domains after cooling specimen from 365 K to 100 K in the absence of magnetic field. (**b-f**) At higher fields, the spiral domains transform to nano-objects spin textures. (**g, h**) Sparse and field polarized states at 498 mT and 540 mT, respectively. The scale bar in (a) corresponds to 1 μm and same for all LTEM images.

Supplementary Figure 3

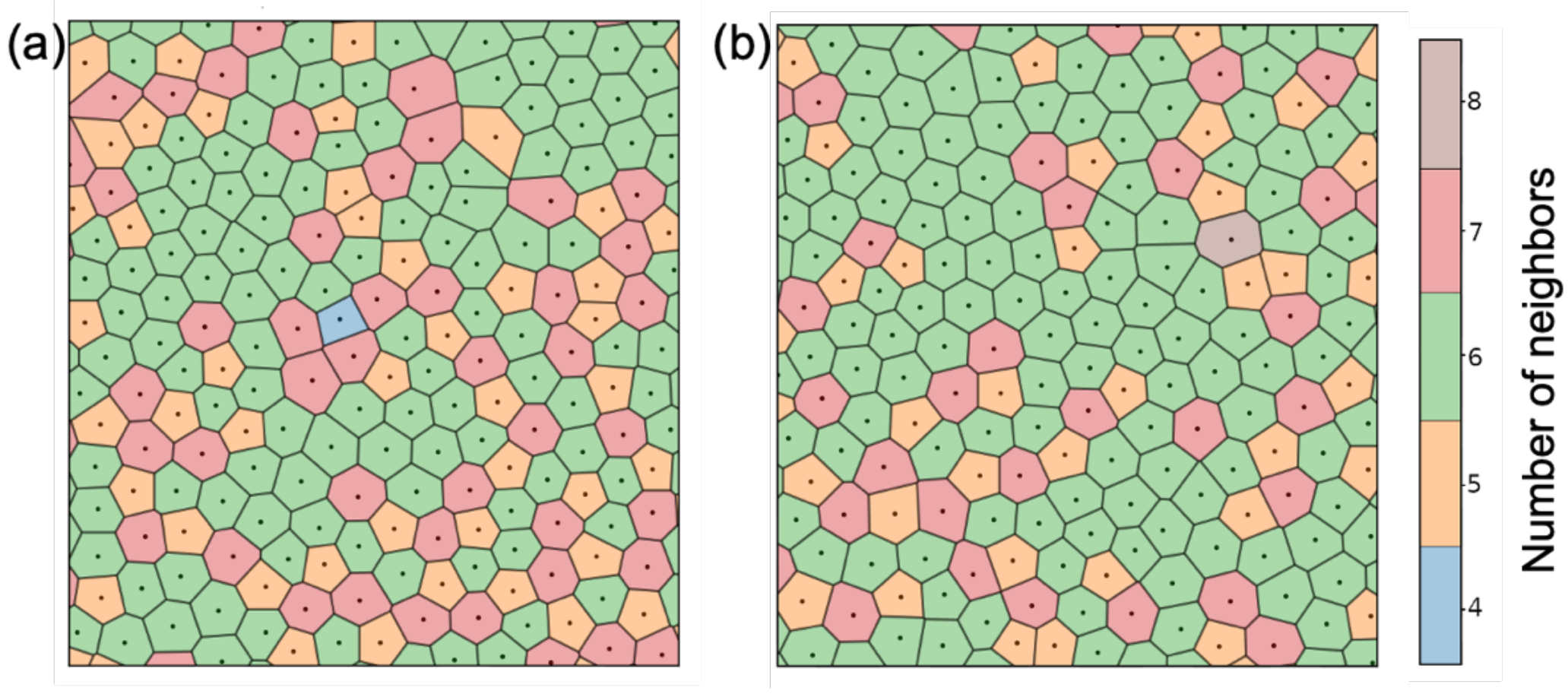

Suppl. Fig. 3: Voronoi diagrams. (**a, b**) Voronoi diagrams show the nearest neighbour objects for the case of field cooling at 15 mT and 51 mT, respectively. We see much more pentagons and heptagons in the Voronoi diagram for the field cooling at 15 mT than at 51 mT.

Supplementary Figure 4

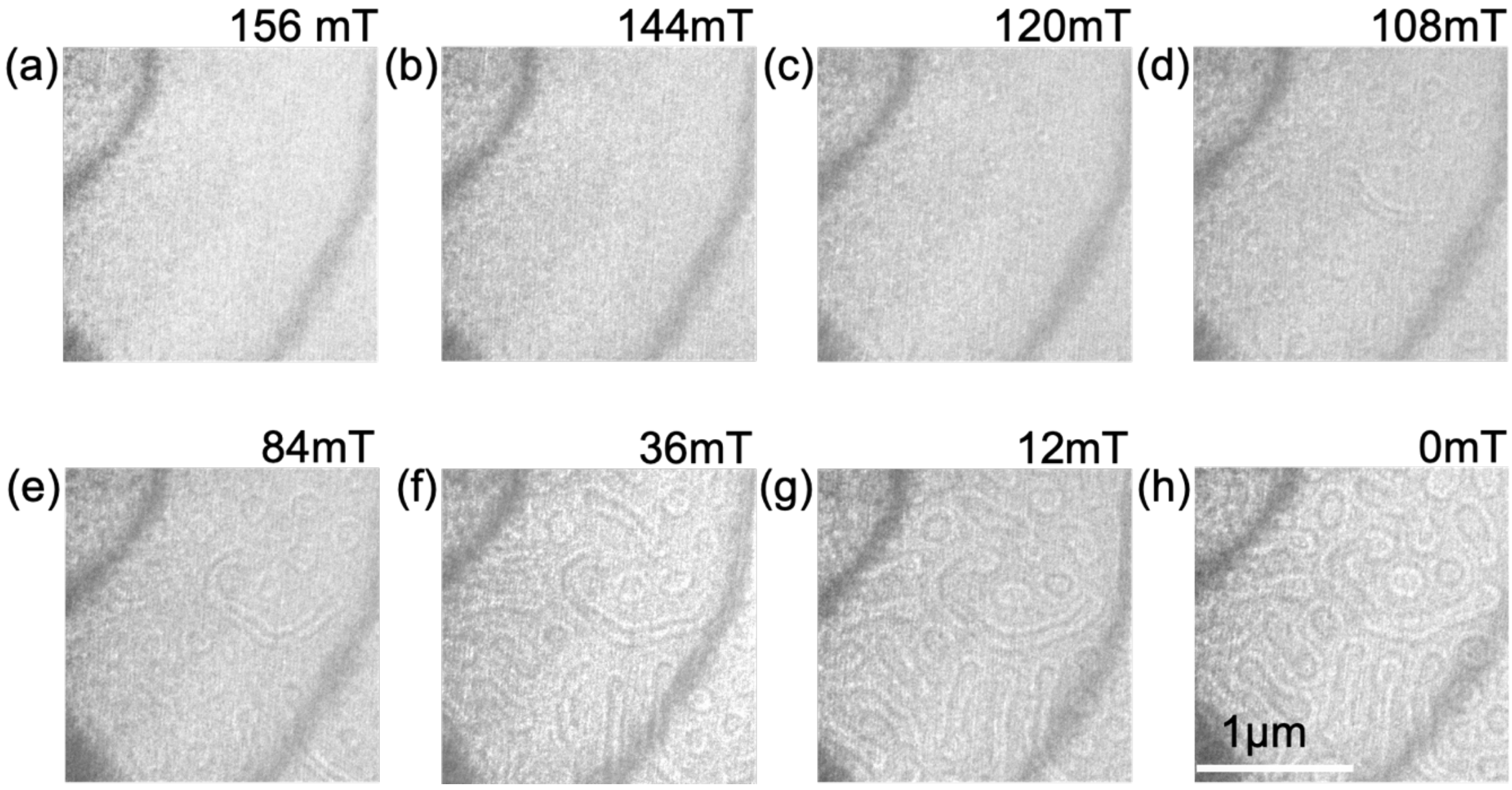

Suppl. Fig. 4: Spin textures evolution starting from polarized state at 323 K. (**a–c**) Upon reducing the magnetic field, a few isolated nano-objects begin to appear around 120 mT. (**d–h**) As the field is further decreased to 0 mT, elongated nano-objects dominate, with only a few circular features remaining. The scale bar in (h) represents 1 μm and is consistent across all LTEM images.

Supplementary Figure 5

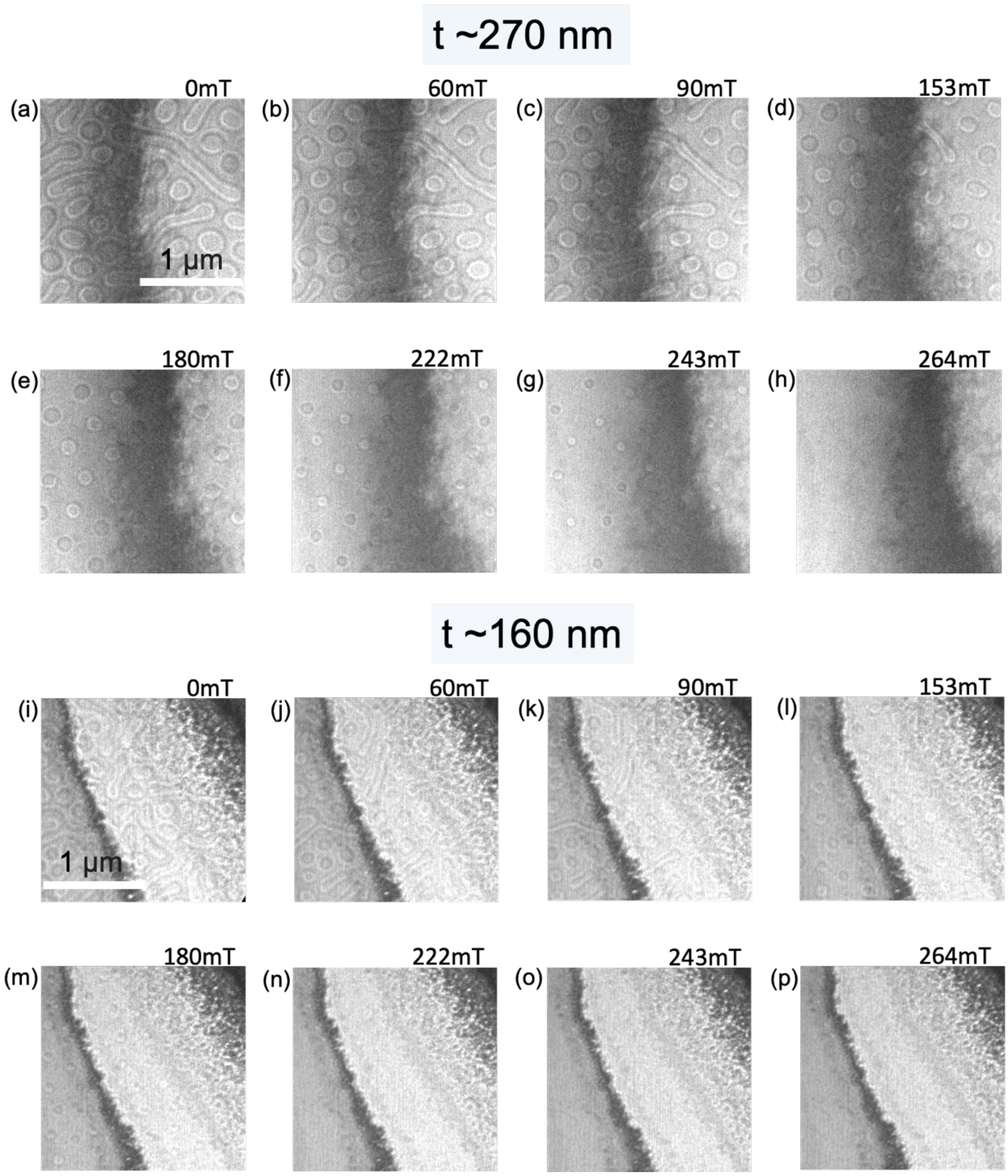

Suppl. Fig. 5: Thickness dependent spontaneous skyrmions and their field driven stability at 300 K. (a) Spontaneous skyrmions and short spirals in the absence of magnetic field for the thickness of 270 nm specimen. (**b-d**) On increasing the out-of-plane magnetic field, the spiral domains transform to nano-objects spin textures. (**e, f**) Nano-object spin textures at 180 mT and 222 mT. (g, h) Sparse and field polarized states at 243 mT and 264 mT, respectively. The scale bar in (a) corresponds to 1 μm and same for all LTEM images. **(i**) Spontaneous skyrmions and short spirals in the absence of magnetic field for the thickness of 160 nm specimen. (**j, k**) On increasing the out-of-plane magnetic field, the spiral domains transform to nano-objects spin textures. (**l**) Nano-object spin textures at 153 mT. (**m**) Sparse state of nano-objects at 180 mT. (**n-p**) Field polarized state at 222 mT, 243 mT and 264 mT, respectively. The scale bar in (i) corresponds to 1 μm and is same for all LTEM images.

Supplementary Figure 6

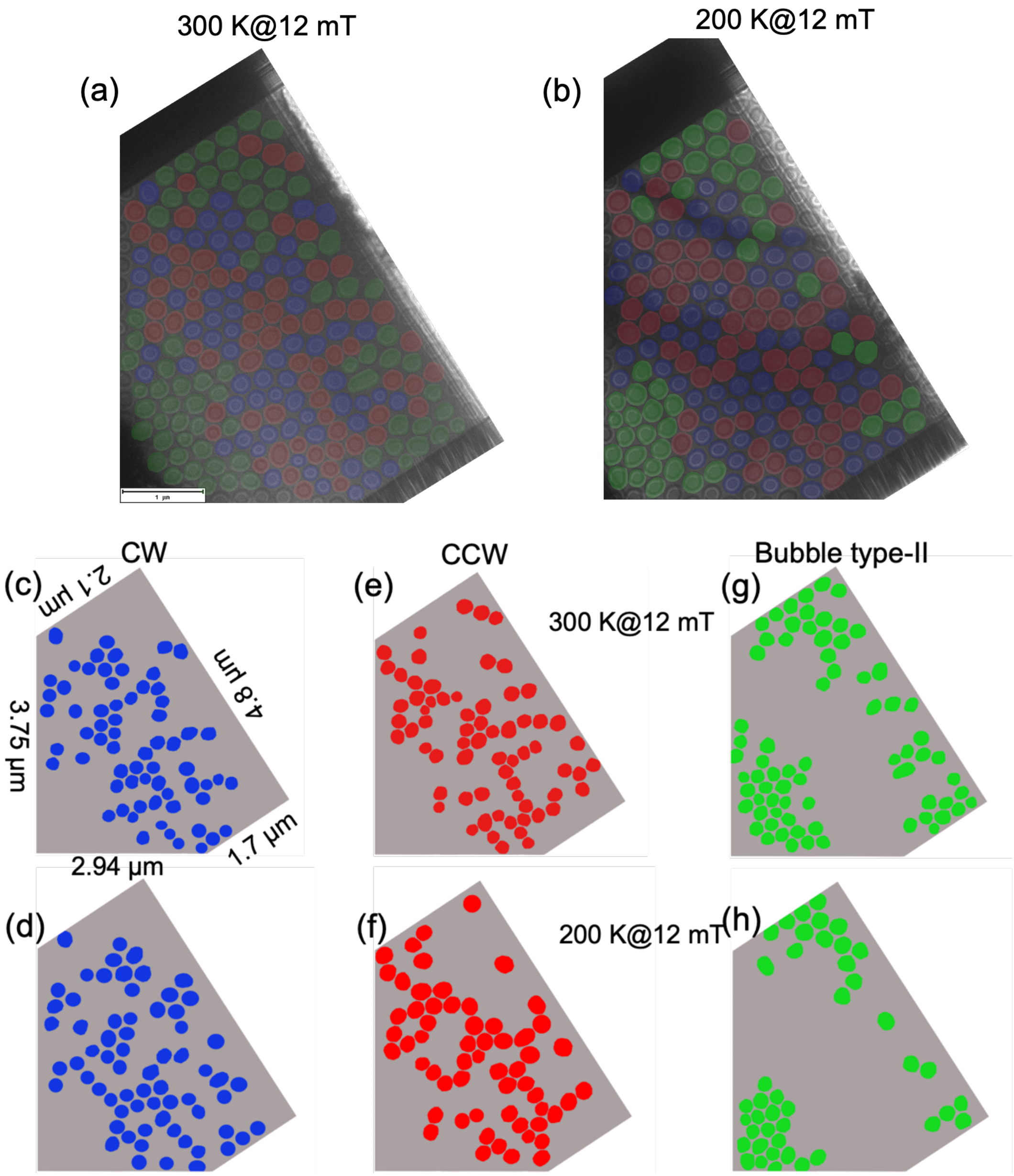

Suppl. Fig. 6: Change in spin textures on field-cooling the 270 nm thick specimen. (a) LTEM contrast for the thicker specimen 270 nm and at 300 K after field cooled the specimen from 365 K and at a constant magnetic field of 12 mT. (**b**) The specimen is cooled to 200 K, keeping the magnetic field constant. (c, d) Clockwise (CW) skyrmions (blue) (e, f) counter-clockwise skyrmions (red), and (g, h) type-II bubbles (green) observed at 300K and 200K under a magnetic field of 12 mT.

Supplementary Figure 7

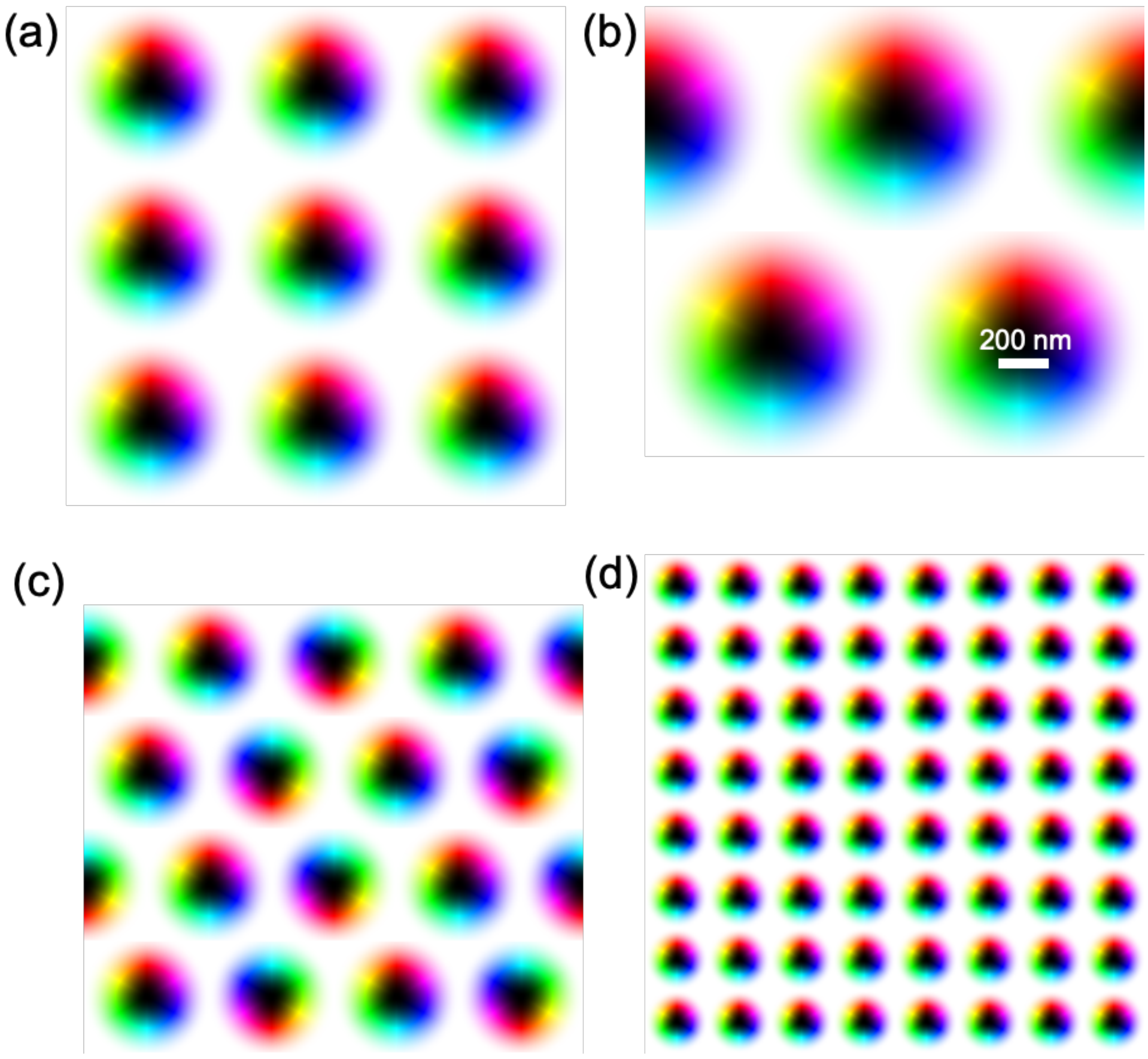

Suppl. Fig. 7: Initial seed configurations used for Fig. 6 in the manuscript. The simulated volume has the dimensions 2000 nm × 1800 nm × 200 nm in Fig. b and c. To avoid finite size effects, the dimension in Fig. a and d are 2000 nm × 2000 nm × 200 nm. The cell size is always 5 nm × 5 nm × 5 nm.

Temperature, and Thickness, and field	CW	CCW	Type II Bubble		Density (total number/μm²)
300 K, 160 nm, 0 mT	19	17	6	Number of skyrmions	5.64
	193.68	213.37	220.20	Diameter in nm	
300K, 200 nm, 0 mT	57	64	28	Number of skyrmions	6.35
	248.01	267.48	253.03	Diameter in nm	
300K, 270 nm, 0 mT	27	25	20	Number of skyrmions	5.47
	256.59	288.45	288.86	Diameter in nm	
300 K, 270 nm, 12 mT	66	66	72	Number of skyrmions	13.86
	234.21	249.94	244.50	Diameter in nm	13.68
200 K, 270 nm, 12 mT	67	59	43	Number of skyrmions	11.48
	249.81	284.62	270.71	Diameter in nm	

Suppl. Table 2: CW and CCW skyrmions in ZFC and FC experiments . Average diameters and number of clockwise (CW) and counter-clockwise (CCW) spontaneous skyrmions for the different thickness of lamellae at zero-field cooled (ZFC) condition and at 300 K. The highest size of skyrmions is observed for 270 nm lamella, followed by 200 nm and 160 nm thin lamellae. The average diameters and numbers of clockwise (CW) skyrmions, counter-clockwise (CCW) skyrmions, and type-II bubbles at 12 mT and at 300 K and 200 K are listed in the last four rows.

Supplementary Figure 8

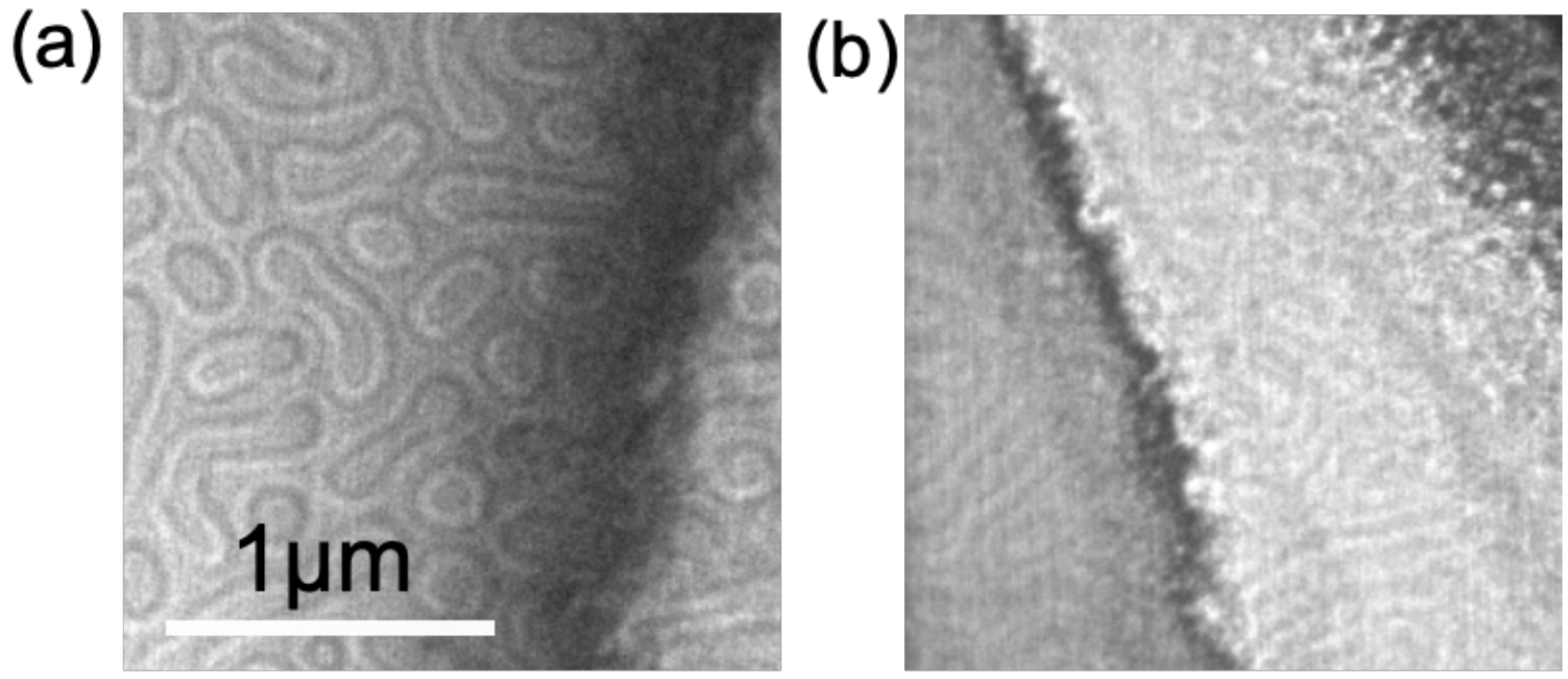

Suppl. Fig. 8:: (a, b) LTEM contrast on inserting the sample at room temperature. The microscope was in LTEM mode. The lamella thickness was around 270 nm and 160 nm for images (a) and (b), respectively. The number of spontaneous skyrmions is slightly higher on cooling the specimen from beyond Curie temperature to the desired temperature because it eliminates the remnant magnetization of the sample as shown in Fig. 2 of the manuscript.

References

1. Moritz, W. & Van Hove, M. A. *Surface Structure Determination by LEED and X-rays*. (Cambridge University Press, 2022).

2. Srivastava, A. K. et al. Observation of robust Néel skyrmions in metallic PtMnGa. *Adv. Mater.* **32,** 1904327 (2020).

3. Chakraborty, A. et al. Magnetic skyrmions in a thickness tunable 2D ferromagnet from a defect driven Dzyaloshinskii–Moriya interaction. *Adv. Mater.* **34,** 2108637 (2022).

4. Sheldrick, G. M. Crystal structure refinement with SHELXL. *Acta Crystallogr. C Struct. Chem.* **71,** 3-8 (2015).

5. Abrahams, S. Indicators of accuracy in structure factor measurement. *Acta Crystallographica Section A: Crystal Physics, Diffraction, Theoretical and General Crystallography* **25,** 165-173 (1969).
