# Supplementary material for: Spontaneous emergence of skyrmions beyond room temperature in boron-doped CrTe
Source: Commun Mater. 2026 Aug 13;7(1):210. doi: 10.1038/s43246-026-01336-1 (PMC13472906; doi:10.1038/s43246-026-01336-1)
Supplement: Supplementary file 2 [file 43246_2026_1336_MOESM2_ESM.pdf]

nature portfolio

Peer Review File

Spontaneous emergence of skyrmions beyond room temperature in boron-doped CrTe

Corresponding Author: Professor Stuart Parkin

This file contains all reviewer reports in order by version, followed by all author rebuttals in order by version.

Version 0:

Reviewer comments:

Reviewer #1

(Remarks to the Author)
In the revised manuscript, Jena and co-workers have appropriately addressed the concerns raised by me and the other reviewers, and have provided more detailed explanations in both the main text and the Supporting Information. Therefore, I basically support the publication of this manuscript in Communications Materials, although I have a few minor comments for the authors' consideration.
The inset in Figure 1a is rather difficult to see. It may be helpful to either enlarge or move it to the Supporting Information, or incorporate it into Figure 1b etc.
For comparison, it would be beneficial to indicate in the phase diagram of Figure 3i the magnetic field corresponding to the saturation magnetization.
The authors collectively define both topologically trivial and non-trivial magnetic bubbles as nano-objects. I still believe that, if possible, it would be preferable to analyze and discuss these two types of objects separately.

Reviewer #3

(Remarks to the Author)
This manuscript “Spontaneous emergence of skyrmions beyond room temperature in boron-doped CrTe” revealed the spontaneous emergence of skyrmions beyond room temperature in a centrosymmetric crystal of boron-doped CrTe without the presence of external magnetic fields. The density and arrangement of metastable zero-field skyrmions via field-cooling process are further confirmed by micromagnetic simulations. The exploration of new materials and results in the skyrmion research field is encouraged. However, there are some improper expressions that should be addressed before publication.
1. There are some additional frames in the figures (Figure1-4) which should not be visible.And the format of images arrange are not good with too much space among images.
2. Scale bar should be put on the image of Figure 2c not diffraction pattern of Figure 2d.
3. The image quality of Figure 6 b-e is not good. It will be better to get a good one.
4. It would make readers confused about the image size change in Figure 5, with the same scale bar but different image size.
5. It makes no sense for the summary in Figure 2i. For example, the skyrmions generated after field cooling would certainly remain due to the increased energy barrier while further decreasing temperature. This manuscript emphasizes the skyrmions beyond room temperature which does not match the marked unexplored region at low temperature. The materials here are only small part of skyrmion materials.
6. Some sentences should be carefully revised for publication. For example, “In summary, we have observed the spontaneous emergence of zero-field Bloch-like skyrmions and type-II bubbles, for the first time in any material, over a wide temperature range from 100 K to beyond room temperature at 332 K, in the absence of any magnetic field.” The spontaneous emergence of zero-field Bloch-like skyrmions and type-II bubbles in wide temperature has been reported in several materials. Check the related references and accordingly revise the introduction part.

Open Access This Peer Review File is licensed under a Creative Commons Attribution 4.0 International License, which permits use, sharing, adaptation, distribution and reproduction in any medium or format, as long as you give appropriate credit to the original author(s) and the source, provide a link to the Creative Commons license, and indicate if changes were made.
In cases where reviewers are anonymous, credit should be given to 'Anonymous Referee' and the source.
The images or other third party material in this Peer Review File are included in the article's Creative Commons license, unless indicated otherwise in a credit line to the material. If material is not included in the article's Creative Commons license and your intended use is not permitted by statutory regulation or exceeds the permitted use, you will need to obtain permission directly from the copyright holder.
To view a copy of this license, visit https://creativecommons.org/licenses/by/4.0/

Reply to Reviewer #1

> In the revised manuscript, Jena and co-workers have appropriately addressed the concerns raised by me and the other reviewers, and have provided more detailed explanations in both the main text and the Supporting Information. Therefore, I basically support the publication of this manuscript in Communications Materials, although I have a few minor comments for the authors' consideration.

We are grateful for the positive feedback and for this recommendation.

> The inset in Figure 1a is rather difficult to see. It may be helpful to either enlarge or move it to the Supporting Information, or incorporate it into Figure 1b etc.

We have followed the referee's recommendation and made it separate figure (Fig. 1b).

> For comparison, it would be beneficial to indicate in the phase diagram of Figure 3i the magnetic field corresponding to the saturation magnetization.

We thank the reviewer for this helpful suggestion. Following the reviewer's recommendation, we have added the magnetic field corresponding to the saturation magnetization at each investigated temperature to the phase diagram in Fig. 3(i). These values are indicated by green star symbols.

> The authors collectively define both topologically trivial and non-trivial magnetic bubbles as nano-objects. I still believe that, if possible, it would be preferable to analyze and discuss these two types of objects separately.

We thank the reviewer for this suggestion. Throughout the manuscript, we distinguish between topologically non-trivial skyrmions and topologically trivial type-II bubbles whenever their individual properties or behaviors are relevant. In particular, both textures are defined and discussed separately on page 5, and their differences are highlighted where appropriate. For the analysis presented in Fig. 3(i), however, our primary objective is to quantify the total population of localized magnetic textures as a function of temperature and magnetic field. Therefore, we collectively refer to skyrmions and type-II bubbles as "nano-objects". This representation is motivated by the fact that both textures occupy comparable areas within the sample and contribute similarly to the overall density of localized magnetic objects. As the magnetic field increases, a type-II bubble may transform into a skyrmion, while the total number of localized magnetic textures remains nearly unchanged. Consequently, separating the two populations would not significantly affect the conclusions drawn from the phase diagram. We agree that the conversion between type-II bubbles and skyrmions is an interesting aspect of the system. For this reason, we discuss the differences between the two textures and their field-driven conversion separately in the manuscript and provide a more detailed analysis in the Supplementary Information (Fig. S6). Since the focus of Fig. 3(i) is the overall evolution and stability of localized magnetic textures rather than the conversion process itself, we believe that the collective representation in terms of "nano-objects" is appropriate for this particular analysis.

Reply to Reviewer #3

This manuscript "Spontaneous emergence of skyrmions beyond room temperature in boron-doped CrTe" revealed the spontaneous emergence of skyrmions beyond room temperature in a centrosymmetric crystal of boron-doped CrTe without the presence of external magnetic fields. The density and arrangement of metastable zero-field skyrmions via field-cooling process are further confirmed by micromagnetic simulations. The exploration of new materials and results in the skyrmion research field is encouraged. However, there are some improper expressions that should be addressed before publication.

We are grateful for the positive feedback and the helpful comments that we discuss below.

1. There are some additional frames in the figures (Figure1-4) which should not be visible. And the format of images arrange are not good with too much space among images.

We are very thankful for this comment and have followed the recommendation to reduce whitespace in our figures. The visible thin frames around some images might occur during Nature's PDF generation/conversion process and are not present in the original figure files. We will ensure that these artifacts are removed and that the final published version contains the high-quality figures.

2. Scale bar should be put on the image of Figure 2c not diffraction pattern of Figure 2d.

We believe the reviewer is referring to Fig. 1(c,d) – now Fig. 1(d,e) in the revised manuscript. The scale bar shown in Fig. 1(d) (now Fig. 1(e)) is not a real-space scale bar but rather a reciprocal-space scale bar associated with the selected-area electron diffraction (SAED) pattern. Therefore, the unit is given in nm^{-1}, which is the standard representation for diffraction data. For the SEM image shown in Fig. 1(c) (Fig. 1(d) in the revised version), the sample dimensions are directly indicated on the image (6.4 μm × 5.9 μm), providing a more precise description than a conventional scale bar. To avoid any confusion, we have revised the figure caption to explicitly state that the scale bar in the diffraction pattern corresponds to reciprocal space and that the SEM image dimensions are directly labeled.

3. The image quality of Figure 6 b-e is not good. It will be better to get a good one.

We thank the reviewer for this comment. We agree that the image quality in Fig. 6(b–e) is lower than that of several other LTEM images presented in the manuscript. However, this difference originates from the sample thickness and the corresponding LTEM imaging conditions rather than from image acquisition or processing. Specifically, the lamella contains regions with substantially different thicknesses. In the thinner region (right side of Fig. 6(b–e)), the integrated magnetic induction along the electron-beam direction is relatively small, resulting in weaker magnetic deflection of the electron beam and consequently reduced LTEM contrast. In contrast, the thicker region (left side of Fig. 6(b–e) and Fig. 6(f–h)) provides a larger magnetic signal, but the increased specimen thickness also reduces electron transmission and degrades image contrast. Therefore, both very thin and very thick regions produce less pronounced LTEM contrast, albeit for different physical reasons. The highest-quality LTEM images

presented elsewhere in the manuscript were obtained from regions with an intermediate thickness of approximately 200 nm, where the magnetic phase shift is sufficiently strong while maintaining adequate electron transmission. Importantly, despite the reduced contrast in Fig. 6(b–e), the magnetic textures remain clearly identifiable and the conclusions drawn from these observations are unaffected.

To clarify this point, we have added an explanation of the thickness-dependent LTEM contrast in the figure caption: *'The variation in image contrast originates from thickness variations within the TEM lamella. Reduced contrast in the thinner region results from a smaller integrated magnetic induction, whereas reduced contrast in the thicker region arises from decreased electron transmission.'*

> 4. It would make readers confused about the image size change in Figure 5, with the same scale bar but different image size.

Indeed, this figure is supposed to be shown only at the width of one text column while many of the other figures a supposed to be shown at full page width in the final version of the paper. We changed the scale bar to 1 μm so that it fits the previous figures and so that there is no confusion.

> 5. It makes no sense for the summary in Figure 2i. For example, the skyrmions generated after field cooling would certainly remain due to the increased energy barrier while further decreasing temperature. This manuscript emphasizes the skyrmions beyond room temperature which does not match the marked unexplored region at low temperature. The materials here are only small part of skyrmion materials.

We follow the suggestion and have removed Fig. 2i.

> 6. Some sentences should be carefully revised for publication. For example, "In summary, we have observed the spontaneous emergence of zero-field Bloch-like skyrmions and type-II bubbles, for the first time in any material, over a wide temperature range from 100 K to beyond room temperature at 332 K, in the absence of any magnetic field." The spontaneous emergence of zero-field Bloch-like skyrmions and type-II bubbles in wide temperature has been reported in several materials. Check the related references and accordingly revise the introduction part.

Following the reviewer's recommendation, we have revised the manuscript to avoid overstating the novelty of the present work. In particular, we have removed claims such as "for the first time in any material" from the Conclusion.
