# Supplementary material for: Spontaneous emergence of skyrmions beyond room temperature in boron-doped CrTe
Source: Commun Mater. 2026 Aug 13;7(1):210. doi: 10.1038/s43246-026-01336-1 (PMC13472906; doi:10.1038/s43246-026-01336-1)
Supplement: Supplementary file 3 [file 43246_2026_1336_MOESM3_ESM.pdf]

Spontaneous emergence of skyrmions beyond room temperature in boron-doped CrTe

Jagannath Jena[1,4,#,*], Konstantin Holst[1,2,#], Börge Göbel[2,#], Yangkun He[3], Binoy Krishna Hazra[1], Pedram Bassirian[1], Katayoon Mohseni[1], Georg Woltersdorf[2], Ingrid Mertig[2], Holger Meyerheim[1], Claudia Felser[3], and Stuart S. P. Parkin[1*]

[1]Max Planck Institute of Microstructure Physics, Halle (Saale), Germany.

[2]Institute of Physics, Martin Luther University Halle-Wittenberg, Halle (Saale), Germany.

[3]Max Planck Institute for Chemical Physics of Solids, Dresden, Germany

[4]Materials Science Division, Argonne National Laboratory, Illinois, USA

#These authors contributed equally.

*Corresponding authors: jjena@anl.gov

stuart.parkin@mpi-halle.mpg.de

Abstract

Magnetic skyrmions are vortex-like spin textures that exhibit remarkable emergent phenomena and hold great promise for applications in spintronics, magnonic devices, and neuromorphic computing. Typically, these spin textures are formed in magnetic samples through the application of magnetic fields on a ground state helical phase. However, achieving the formation of zero-field skyrmions, without the need of prior field-cooling, has remained a significant challenge. Here, using real-space Lorentz transmission electron microscopy, we directly observe the spontaneous emergence of skyrmions beyond room temperature in a centrosymmetric crystal of boron-doped CrTe without the presence of external magnetic fields. These skyrmions exhibit a robust stability over a large temperature range in zero field and various specimen thicknesses. We nevertheless demonstrate that a field-cooling process can control the density and arrangement of metastable zero-field skyrmions which is confirmed by micromagnetic simulations. The stabilization of field-free spontaneous skyrmions beyond ambient temperature holds implications for nonvolatile memory systems.

Introduction

Nanoscopic spin textured objects, including magnetic domain walls and skyrmions, are sought after as next-generation information storage units for use in magnetic memory-storage technologies, due to their high stability, variable size, and especially their ability to be manipulated by current.[1-4] Skyrmions are 2D and 3D objects that typically have complex spin textures delineated by 2D or 3D boundaries (akin to domain walls) in a host magnetic material. The actual structure is realized by the competition between distinct magnetic interactions. Skyrmions have been observed in a variety of magnetic materials primarily non-centrosymmetric systems but, in rare cases, centrosymmetric systems. In the former case, the formation of chiral skyrmions is primarily attributed to an asymmetric Dzyaloshinskii-Moriya vector exchange interaction (DMI)[5, 6] that appears in systems with broken inversion symmetry (examples of crystallographic point groups which fulfill this requirement are T[4, 7], O[8], C_{nv}[9], D_{2d}[10, 11], and S_4[12]). Conversely, in centrosymmetric systems the interplay of magnetic anisotropy and dipolar interactions has been shown to allow for the generation of topologically non-trivial Bloch skyrmions with positive and negative helicities ($\pm\pi/2$), as well as topologically trivial type-II bubbles.[13-17] In many of these magnetic materials, the magnetic ground state consists of helices or stripe domains, while skyrmions are observed only in the presence of an externally applied magnetic field. Thus far, stabilizing skyrmions in zero magnetic field has been challenging and typically requires complex conditioning, such as geometrical confinement[18, 19], sweeping an external magnetic field from a non-zero field that allows for a skyrmion lattice state[20] or cooling the sample to low temperatures in a magnetic field and then removing the field.[21-25] Potential applications of skyrmions are limited by the necessity of an external magnetic field, which makes such devices much more complex.

It has been shown theoretically that non-centrosymmetric metallic magnets may spontaneously produce a skyrmion ground state in the absence of a magnetic field.[26, 27] However, to date there have only been a small number of experimental examples, that include monolayers of Fe and Co on Ir(111) albeit at extremely low temperatures and in which the center of symmetry is broken interfacially.[28, 29] Recently, zero-field biskyrmions were observed in two Nd-Co based rare-earth compounds[30, 31]. Additionally, zero-field skyrmions have also been reported to form due to edge instabilities in the helical state under the application of current, stress, or the exchange bias effect[32-34].

In this study, we present the emergence of spontaneous skyrmions in the compound $Cr_{0.9}B_{0.1}Te$ beyond room temperature stabilized without any magnetic field. Real-space Lorentz transmission electron microscopy (LTEM) imaging directly visualizes these zero-field skyrmions which coexist with short stripe domains and which exhibit remarkable stability over a wide temperature range between 100 K (the lowest accessible temperature in our microscope) and 332 K. Moreover, we demonstrate that the application of a small magnetic field leads exclusively to the formation of the skyrmion state without the presence of short stripe domains when cooling from a temperature above the critical temperature T_c of the compound down to ambient temperature. Intriguingly, the rather dense skyrmion lattice remains stable even at zero field. We elucidate the role of dipole-dipole interactions in the stabilization of these zero-field skyrmions by comparing results for different sample thicknesses. Our findings shed light on the complex behavior of skyrmion formation in this material and opens new avenues for exploring their properties and potential applications.

Results and discussion

Single crystals of $Cr_{0.9}B_{0.1}Te$ were prepared as described in reference[35]. X-ray diffraction (XRD) analysis of our samples reveals that the samples crystallize in the trigonal centrosymmetric space group (SGR) $P\bar{3}2/m$ (164) rather than the previously reported hexagonal centrosymmetric SGR $P6_3/mmc$ (194). The crystal structure closely resembles the latter structure (NiAs), but we find an additional small fraction of Cr atoms (site occupancy of approximately 10%) that reside close to the tetrahedrally coordinated sites (Wyckoff site 2d($^2/_3$, $^1/_3$, 0.35) in SGR 164) above and below the Te atoms. Further details of the structural analysis are discussed in the Supplemental Information (Suppl. Fig. 1 and Suppl. Table 1). Out-of-plane and in-plane magnetization, M, vs. temperature, T, curves of the bulk material (Fig. 1a) were measured using a superconducting quantum interference device (SQUID) magnetometer and showed a paramagnetic to ferromagnetic transition T_c at 335±1 K, which is also reflected in ac susceptibility measurements, as presented in Fig. 1b. An additional kink appears in both measurements at a lower temperature of ~138 K, that has been shown in bulk crystals of $Cr_{0.9}B_{0.1}Te$ to be a spin reorientation transition temperature (T_{SRT}), indicative of a non-collinear spin structure that forms below this temperature.[35] At 325 K the magnetization corresponds to 1.1 μ_B/f. u. reaching 2.8 μ_B/f. u. at 2 K (Fig. 1c). Ga^+-focused ion beam (FIB) milling was used to prepare thin lamellae from the bulk crystals with the [001] axis oriented perpendicular to the

lamella. Using this technique, a homogenous specimen with a thickness of ~200 nm was fabricated. Fig. 1d displays a scanning electron microscopy (SEM) image of the specimen's surface. Fig. 1e shows a selected area electron diffraction (SAED) pattern from transmission electron microscopy (TEM) studies, which confirms the [001] orientation of the specimen. Magnetic imaging from the thin specimens was performed using LTEM. LTEM imaging (i) can distinguish Bloch skyrmions[7], Néel skyrmions[36], topologically trivial bubbles[15], antiskyrmions[10] and other exotic spin textures[20, 37, 38], (ii) has a spatial resolution of ~10 nm, and (iii) has a relatively short acquisition time compared to other imaging techniques.[39, 40] The specimens were placed in a liquid nitrogen (LN_2) based holder in which the temperature could be varied between 100 K and 365 K.

The absence of a DMI in a centrosymmetric trigonal structure and the presence of dipole-dipole interactions give rise to two types of Bloch skyrmion bubbles each with a topological charge $\boldsymbol{N_{\mathrm{Sk}}} = \frac{\mathbf{1}}{\mathbf{4\pi}} \iint \mathbf{m(r)} \cdot \left(\frac{\partial \mathbf{m(r)}}{\partial \boldsymbol{x}} \times \frac{\partial \mathbf{m(r)}}{\partial \boldsymbol{y}} \right) \mathbf{d^2 r} = \mathbf{-1}$, as well as topologically trivial type-II bubbles for which $N_{\mathrm{Sk}} = 0$. Micromagnetic simulations of these nano-objects, namely the clockwise (CW) Bloch skyrmion, the counter-clockwise (CCW) Bloch skyrmion, and the type-II bubble, are depicted in Fig. 1f-h, respectively. Simulations of the corresponding LTEM images and those observed experimentally match each other very well. Note that the opposite helicity of the spin textures of the CW and CCW Bloch skyrmions are reflected by the inverted LTEM contrast of the respective images. For the case of the topologically trivial type-II bubble, Bloch walls with opposite helicities are present, separated by Bloch lines (Néel-like walls).

To systematically investigate the formation and stability of skyrmions, the temperature of the specimen was first increased to $T = 365$ K > T_c. On cooling in the absence of a magnetic field, a magnetic LTEM contrast is not observed until 325 K, where spontaneous skyrmions can be seen along with elongated objects (Fig. 2b). The LTEM images recorded on further cooling to 100 K are similar although with increasing contrast as the magnetization systematically increases (see Fig. 2c-h). From the topological point of view, the elongated spin textures denoted by the orange and purple loops in Fig. 2f are presumably characterized by a topological charge that is the same as that of the Bloch skyrmions. For this reason, they can be considered elongated Bloch skyrmions[41]. A few other elongated textures have both CW and CCW Bloch walls (indicated by the orange and purple lines in a single nano-object) and are therefore topologically trivial. They are equivalent to elongated type-II bubbles.

In most previous reports, it has been demonstrated that zero-field skyrmions exist as a metastable state in a variety of materials.[18-22, 42] This means that the skyrmions can only be stabilized at zero field after undergoing a prior field-cooling treatment. Here we show that skyrmions in boron-doped CrTe spontaneously form without the need of a magnetic field cooling protocol and that these skyrmions are stable well above room temperature.

The magnetic field evolution of the spontaneous magnetic nanostructures found in $Cr_{0.9}B_{0.1}Te$ was investigated by applying an out-of-plane external magnetic field. The circular and elongated objects observed at zero magnetic field are shown in Fig. 3a. The size of the elongated objects and skyrmions decreases as the magnetic field is increased, as shown in Fig. 3b and 3c. At 144 mT (Fig. 3d) and 162 mT (Fig. 3e), the elongated objects shrink in such a way that they form circular nano-objects that are either trivial type-II bubbles or Bloch skyrmions, depending on the initial state of the elongated object. At 192 mT (Fig. 3f), a lattice state develops, even though the periodicity is not perfect. As shown in Fig. 3g the density of nano-objects decreases, resulting in a sparse lattice state at 234 mT. With a further increase of the magnetic field to 240 mT (Fig. 3h), the field-polarized state is formed, with all moments aligned along the applied field direction so that the LTEM image contrast vanishes.

To investigate the temperature evolution of skyrmions, the above experiment was repeated at various temperatures and a magnetic phase diagram was constructed, as depicted in Fig. 3i. The color code in the diagram represents the density of the observed nano-objects including Bloch skyrmions of both helicities and topologically trivial bubbles. As evidenced in the phase diagram, the field stability region of the skyrmions increases with decreasing temperature. The field evolution of the spin textures at 100 K is shown in Suppl. Fig. 2 which shows that the field stability region is approximately twice as large as at 300 K.

So far, all experiments have been conducted following a zero-field cooling protocol for the initial formation of the magnetic state. Next, we perform field-cooling (FC) experiments to allow for different magnetic configurations that may arise due to the metastability of the nano-objects in our sample (Fig. 4). Initially, the specimen was heated to $T = 365$ K $> T_c$, followed by the application of a finite magnetic field at that temperature. The specimen is then cooled to ambient temperature while maintaining the constant magnetic field. We repeated this FC method using different strengths of the magnetic field. Figs. 4a-f show the LTEM contrasts after FC to ambient temperature under various magnetic field strengths. At 6 mT (Fig. 4a,) the result is similar to our previous observations at 0 mT with both circular and elongated nano-

objects. When a higher magnetic field strength is applied during the FC process, the density of nano-objects becomes larger. The elongated objects disappear above 15 mT (Fig. 4b and 4c). In the latter case, the skyrmions and topologically trivial bubbles are smaller. With further increasing field during the FC process, the density of all nano-objects decreases, as observed for 66 mT (Fig. 4d) and 81 mT (Fig. 4e), and eventually, a field-polarized state is observed at 120 mT (Fig. 4f). Besides these findings, we noticed that a greater proportion of topologically trivial bubbles are formed at higher field strengths whereas the proportion of Bloch skyrmions is higher at lower field strengths during FC.

After the individual magnetic configurations have been stabilized under the above explained FC protocol with distinct magnetic field strengths, we compare how they change when the magnetic field is reduced to zero after their formation (Fig. 4g-l). For the configurations that have initially been formed under FC between 6 mT and 66 mT, the nano-objects remain stable (Fig. 4g-j) and we observe no notable change in the magnetic textures. In these cases, the skyrmion density is comparably high. However, for the configuration that was field-cooled at 81 mT, the number of non-collinear objects in the sample is lower and a reduction of the magnetic field to zero leads to size changes of several magnetic textures (Fig. 4k). For the ferromagnetic state that appears under field cooling at 120 mT, the region is filled only with spirals in the absence of a magnetic field (Fig. 4l).

These results point out the importance of the experimental protocol for the formation of non- collinear spin textures. All the presented images in Fig. 4g-l have been recorded under exactly the same conditions, but the sample hosts different numbers and types of spin textures caused by the distinct experimental histories. While applying a magnetic field under the cooling process from 365 K to room temperature is generally not required for the formation of bubbles and skyrmions, their nucleation can be fostered, and their density increased when a finite magnetic field is applied. The ideal FC field for that was ~51 mT in our experiments.

Continuing from the distinct zero-field states shown in Fig. 4g-l, the magnetic field is now increased to 192 mT (Fig. 4m-r). In all cases, the number of stabilized nano-objects remains almost the same and the size of the nano-objects decreases compared to the state at 0 mT. Still, major changes are observed, most importantly for the measurement that started with FC at 81 mT (Fig. 4e). Here the shrinking of the textures causes the initially elongated objects to form almost perfectly circular skyrmionic bubbles. As before, the various configurations

recorded at the same field of 192 mT are very different from each other due to the experimental history.

Fig. 4s summarizes and generalizes the above discussed results. The plot depicts the number of nano-objects (skyrmions and bubbles) that we have observed for different values of the applied magnetic field. The individual curves differ only by their starting configuration which were generated under different magnetic field strengths applied during field cooling. These configurations correspond to Figs. 4a-f, as indicated. As mentioned before, the density of nano-objects shows an increasing trend with the magnetic field that was applied during field-cooling up to a certain strength and drops for higher values. For the state that was stabilized under a field of 51 mT, at which we see the largest number of nano-objects, many of those have 6 neighbors. A nearly periodic, hexagonal lattice is formed (see Suppl. Fig. 3). However, the further we go away from this field during FC, the fewer nano-objects we observe and the more random the ordering appears. Once the saturation state is achieved, reducing the magnetic field typically leads to the formation of a labyrinthine domain state, occasionally accompanied by a few isolated nano-objects embedded within it. This behavior is consistent across all low temperatures, except near Tc. Near Tc, we observe a distinct response: nano-objects emerge directly during the field reduction from the polarized (saturated) state, as illustrated in Suppl. Fig. 4.

The experimental results indicate a manifold of allowed metastable configurations. The stabilized magnetic state can be controlled by the precise experimental protocol. In the following, we model this process by micromagnetic simulations (at zero temperature) and show that the stabilized configuration strongly depends on the starting configuration which acts as a seed. Note that periodic boundary conditions are imposed. The seed emulates the different field-cooling conditions from Fig. 4. We have used the specific material parameters of the measured sample. Technical details about the simulations are presented in the Methods section. As shown in Fig. 2, zero-field cooling leads to a mixed magnetic state consisting of circular and elongated skyrmionic bubbles of both helicities as well as topologically trivial bubbles. This process can be emulated in micromagnetic simulations by starting the simulation with a seed that contains only a few skyrmions. For example (Fig. 5a), nine skyrmions turn into a metastable state exhibiting five skyrmionic bubbles and four elongated magnetic textures. When the skyrmion number in the seed is decreased to four (Fig. 5b), the result is a configuration consisting of four elongated textures. This is similar to the experimental scenario

of field-cooling under a rather large magnetic field (like in Fig. 4e, FC 81 mT). Once the field has been turned off, mostly helices are present.

In the experiments, a dense skyrmion lattice can be stabilized under field-cooling at 51 mT as presented in Fig. 4c. In the simulation this state is also stable; see Fig. 5c. If the seed is chosen to be a periodic lattice of 16 skyrmions, the configuration remains metastable, and we even observe both skyrmion helicities. Topologically trivial bubbles are unstable which could be related to the fact that our simulations have been carried out without taking thermal effects into account. Note that in the experiments, skyrmions become more stable than topologically trivial bubbles at low temperatures as well.

When we start with an even higher number of skyrmions in the simulations, here 36, the individual skyrmions are too small to be stable. Some of these skyrmions spontaneously disappear but others merge, so that the number of skyrmions in the final stable configuration is reduced (here to 20) (Fig. 5d). This state is not periodic and the individual skyrmions have different sizes and shapes, just like the experimental results e.g., in Fig. 4. The magnetic texture is trapped in a local energy minimum and cannot achieve the periodic lattice state.

Lastly, we investigate the thickness dependence of the skyrmions and topologically trivial bubbles. We carried out similar experiments on a second lamella that has two regions with thicknesses of 160 nm and 270 nm (Fig. 6a) compared to the thickness of 200 nm that was used before. In this lamella, we also observe the emergence of spontaneous skyrmions at various temperatures at and beyond room temperature (Fig. 6b-e). Therefore, it is reasonable to assert that the formation of spontaneous skyrmion spin textures is inherent in the material. Interestingly, the thicker region exhibits spontaneous magnetic contrast even at 332 K, whereas the thinner region does not. This could be caused by a slightly different energy landscape due to an increased importance of the dipolar interactions. For this reason, we also see that the size of the skyrmions increases as the thickness of the specimen increases (See Table 2 in SI info.). Furthermore, we find that the field stability region of nano-objects for thicker specimens is larger than for thinner ones. This is presented in the Suppl. Fig. 5. However, another possibility for why we do not see any magnetic contrast in the thinner region at 332 K could be the smaller magnetic induction compared to the thicker region that deflects the transmitted electron beam less after passing through the specimen. Therefore, even though the compound has very low moment close to T_c, skyrmionic spin textures can still be observed in the thicker region. In

agreement with this argument, the thinner region generally shows a weaker magnetic contrast than the thicker region (Figs. 6c-e).

In the thicker region of the sample, we also observe conversion processes when the specimen was cooled to a low temperature from 300 K under a constant magnetic field. As seen in Figs. 6f–h, a few trivial bubbles convert to Bloch skyrmions and some Bloch skyrmions change their helicity (CW to CCW and vice versa). The red and green arrows mark the conversion of trivial bubbles to CW Bloch skyrmions and CCW Bloch skyrmions, respectively, when cooling from 300 K to 200 K. Additionally, the blue arrows highlight the conversion of CW Bloch skyrmions at 300 K to CCW Bloch skyrmions at 200 K, whereas the purple arrows indicate the reverse conversion process. Moreover, a few of the nano-objects vanish at 200K, causing the neighboring objects to expand in size (Suppl. Fig. 6). As discussed above, the magnetic objects try to fill the available space as much as possible. There were no such changes in spin textures when the specimen was cooled from 200 K to 100 K.

Conclusion

In summary, we have observed the spontaneous emergence of zero-field Bloch-like skyrmions and type-II bubbles, over a wide temperature range from 100 K to beyond room temperature at 332 K, in the absence of any magnetic field. We have shown that these objects are stabilized by dipole-dipole interactions in $Cr_{0.9}B_{0.1}Te$, a centrosymmetric material and, thus, without any intrinsic DMI. The skyrmions coexist with a few topologically trivial bubbles and elongated objects where the detailed magnetic configuration depends strongly on the experimental protocol used. The density of the skyrmions and whether they form a lattice state depends on the field-cooling procedure. Unlike DMI-stabilized skyrmions, these skyrmions and bubbles are rather flexible, and their density can be varied greatly. The field cooling procedure can be used to control the nucleation rate of the skyrmions. Depending on the number of nano-objects in the sample, they are circular in the preferred magnetic field range but they elongate and fill up all the available space once the field is absent because they try to turn into spiral domains. This is confirmed by micromagnetic simulations by means of different seed configurations that lead to different metastable magnetic configurations once relaxed. The discovery of spontaneous skyrmions in the crystal $Cr_{0.9}B_{0.1}Te$ occurring without any external field or geometric constraint, contributes significantly to our understanding of skyrmion

physics. This new understanding will assist the analysis of the 3D magnetization of skyrmions without the need for complex experimental setups because these imaging techniques will not be disturbed by the need for magnetic fields.[43] Moreover, the spontaneous coexistence of skyrmions with elongated nano-objects might serve as a reservoir with enhanced nonlinear functionalities in the realm of reservoir computing. All desirable features for the application of skyrmions, such as a wide temperature range, high density, zero magnetic field, and thickness-independent stability, were simultaneously observed in the same material in our study, widening the applicability and opening exciting avenues for skyrmion physics.

Methods

Lamella preparation

Single crystalline $Cr_{0.9}B_{0.1}Te$ specimens were produced from the [001] oriented surface from the bulk crystal by using Ga^+ ion based dual beam focused ion beam (FIB) system [FEI Nova Nanolab 600 SEM/FIB]. The FIB is operated at an accelerating voltage of 2-30 kV. The final smooth polishing of the specimen is carried out at 2 kV and 4.4 pA. Scanning electron microscope (SEM) imaging inside the FIB system was used to measure the thickness of the specimens under a zero-tilt stage condition and the dimensions of the prepared specimen was determined from SEM imaging at 52° normal to the surface.

X-ray diffraction

The structural analysis of the $Cr_{0.9}B_{0.1}Te$ crystal was carried out using a six-circle diffractometer and a Gallium-Jet x-ray source (λ = 1.3414 Å) operated at 70 keV and 200 W power. The beam was focused onto the sample by Montel optics providing a highly collimated beam of 100 μm height and 2 mm width. Integrated reflection intensities were collected using transverse phi-scans of the sample and a two-dimensional pixel detector.

Transmission electron microscopy and analysis of the magnetic nanostructure

The SAED and LTEM measurements were performed in a high-resolution transmission electron microscope [JEOL JEM-F200]. A 100 μm selected area aperture was used to capture the diffraction pattern of the single crystalline specimens. A beam stopper is used to block the high intensity (000) central diffraction spot. Magnetic domain contrasts were acquired in Fresnel mode (LTEM mode) with a negligible remnant magnetic field (< 2 mT). The magnetic field was varied along the microscope axis by exciting the objective lens. A double tilt liquid nitrogen holder was used to adjust the specimen's temperature, and a temperature controller was used to maintain the temperature of the specimens at varied levels. The specimens are not tilted at any point during the cooling at any temperature, magnetic imaging or applied magnetic field. The over-focus value for the LTEM imaging is ~0.7-0.9 mm.

Micromagnetic simulations

We have used the GPU-accelerated micromagnetic code mumax3 to stabilize the different magnetic configurations in Fig. 6. The corresponding seed magnetizations (Suppl. Fig. 6) have been constructed from circular Bloch skyrmions that are positioned in a sample with periodic boundary conditions along x and y to emulate an extended sample. The simulated volume has the dimensions 2000 nm $\times$ 1800 nm $\times$ 200 nm in Fig. 6b and 6c. To avoid finite size effects, the dimensions in Fig. 6a and 6d are $2000\,\mathrm{nm} \times 2000\,\mathrm{nm} \times 200\,\mathrm{nm}$. A cell size of 5 nm $\times$ 5 nm $\times$ 5 nm was used. Values of saturation magnetization of $M_s = 3.42 \times 10^5$ A/m, exchange $A = 2.2 \times 10^{-11}$ J/m, and anisotropy $K_z = 0.4 \times 10^5$ J/m^3 were used. Compared to Liu et al,[44] we have measured a significantly smaller uniaxial anisotropy K_z (their value $K_z = 0.79 \times 10^5$ J/m^3). This leads to a larger bubble size and especially to larger domain walls in the bubbles in both the experiment and the micromagnetic simulations. For the relaxation we have used the ‘relax’ command based on energy minimization and a RK23 method (Runge-Kutta method), followed by a 50 ns propagation according to the Landau-Lifshitz Gilbert equation and a RK45 method. The seed configurations are shown in the supplementary material (Suppl. Fig. 7).

Acknowledgements

We acknowledge funding from the European Research Council (ERC) under the European Union’s Horizon 2020 research and innovation program (grant agreement SORBET No. 670166) and the Deutsche Forschungsgemeinschaft (DFG, German Research Foundation)—Project number 403505322 under SPP2137. C.F. acknowledges funding by the DFG under SPP 2137 (Project number 403502666). I.M. and B.G. acknowledge funding by the EIC Pathfinder OPEN Grant No. 101129641, “Orbital Engineering for Innovative Electronics.”.

Author contributions

J.J. and S.S.P.P. conceived the study. J.J. carried out the Lorentz TEM observations and J.J. and K.H. analyzed Lorentz TEM data. The bulk material was synthesized by Y.H. and C.F.. J.J. and K.H. prepared the lamellae of different thicknesses. B.G. and K.H. performed the micromagnetic simulations. P.B., I.M., G.W. helped with the analysis of the manuscript results. J.J. and B.K.H. performed the magnetization measurements. H.M. and K.M. contributed to the crystal structure analysis. J.J., K.H., B.G., and S.S.S.P. wrote the manuscript with substantial contributions from all authors. S.SP.P. supervised the project.

Data availability statement

The data that support the findings of this study are available from the corresponding authors upon reasonable request.

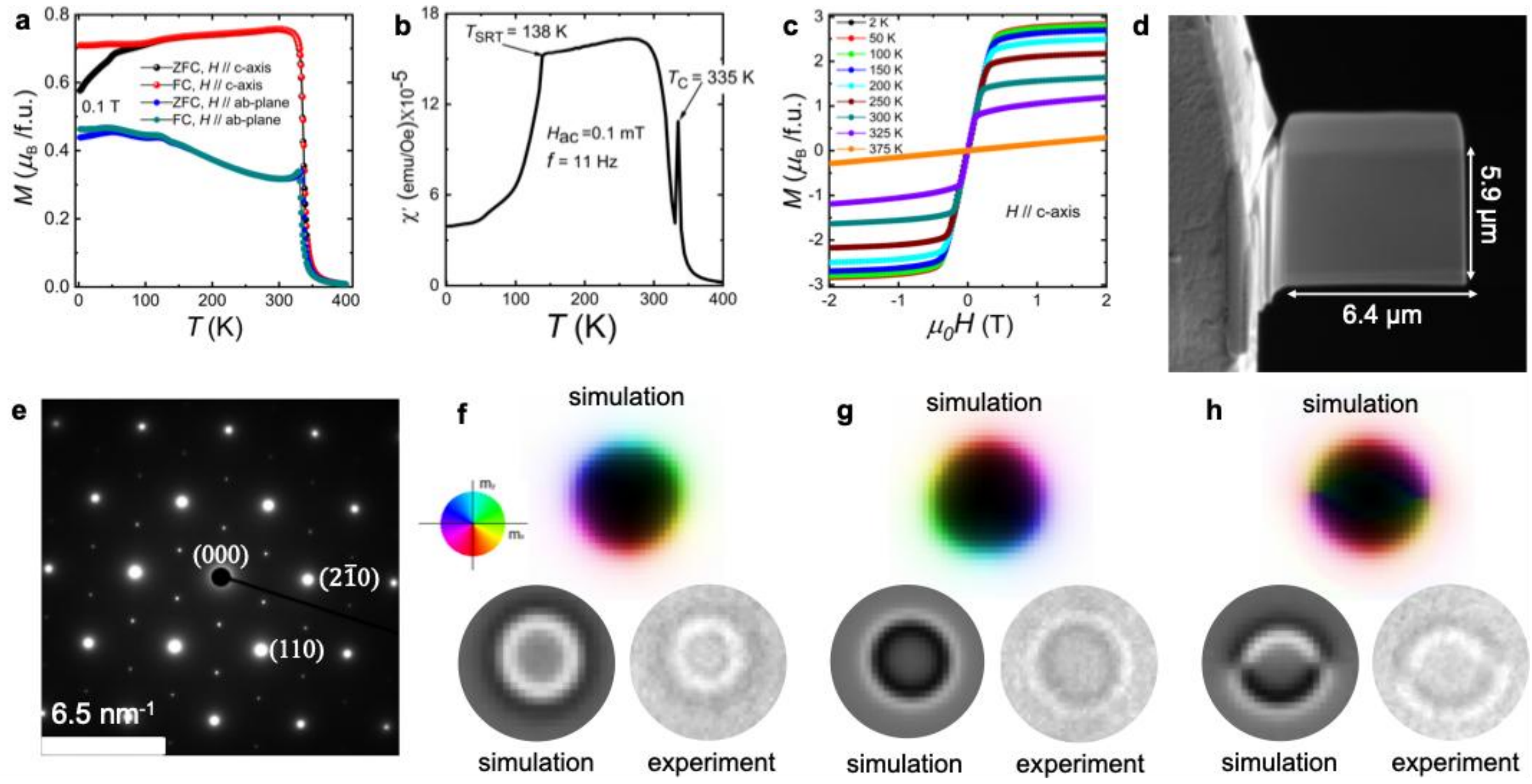

Fig. 1 | Crystal structure and spin textures in $Cr_{0.9}B_{0.1}Te$.

a In-plane ($H \parallel ab$-plane) and out-of-plane ($H \parallel c$-axis) magnetization vs. temperature of the bulk sample for a magnetic field with strength 0.1 T. **b** Shows the ac susceptibility at 11 Hz and 0.1 mT ac magnetic field. **c** Out-of-plane magnetization vs. magnetic field of the bulk sample at various temperatures. **d** A thin specimen is lifted out from the bulk single crystal by using a focused ion beam (FIB) milling technique. Scanning electron microscopy SEM image shows the height and width of the specimen are 5.9 μm and 6.4 μm, respectively. **e** Selected area electron diffraction (SAED) image of the specimen. The scale bar in the diffraction pattern corresponds to reciprocal space and that the SEM image dimensions are directly labeled. **f-h** The upper panel shows the micromagnetically simulated spin textures of a clockwise Bloch skyrmion, counter-clockwise Bloch skyrmion and type-II topologically trivial bubble, respectively. The lower panel shows the LTEM simulations (sim.) of the corresponding textures and experimental (expt.) LTEM images that are observed in a thin specimen of $Cr_{0.9}B_{0.1}Te$. The color wheel in (e) represents the in-plane magnetization distributions.

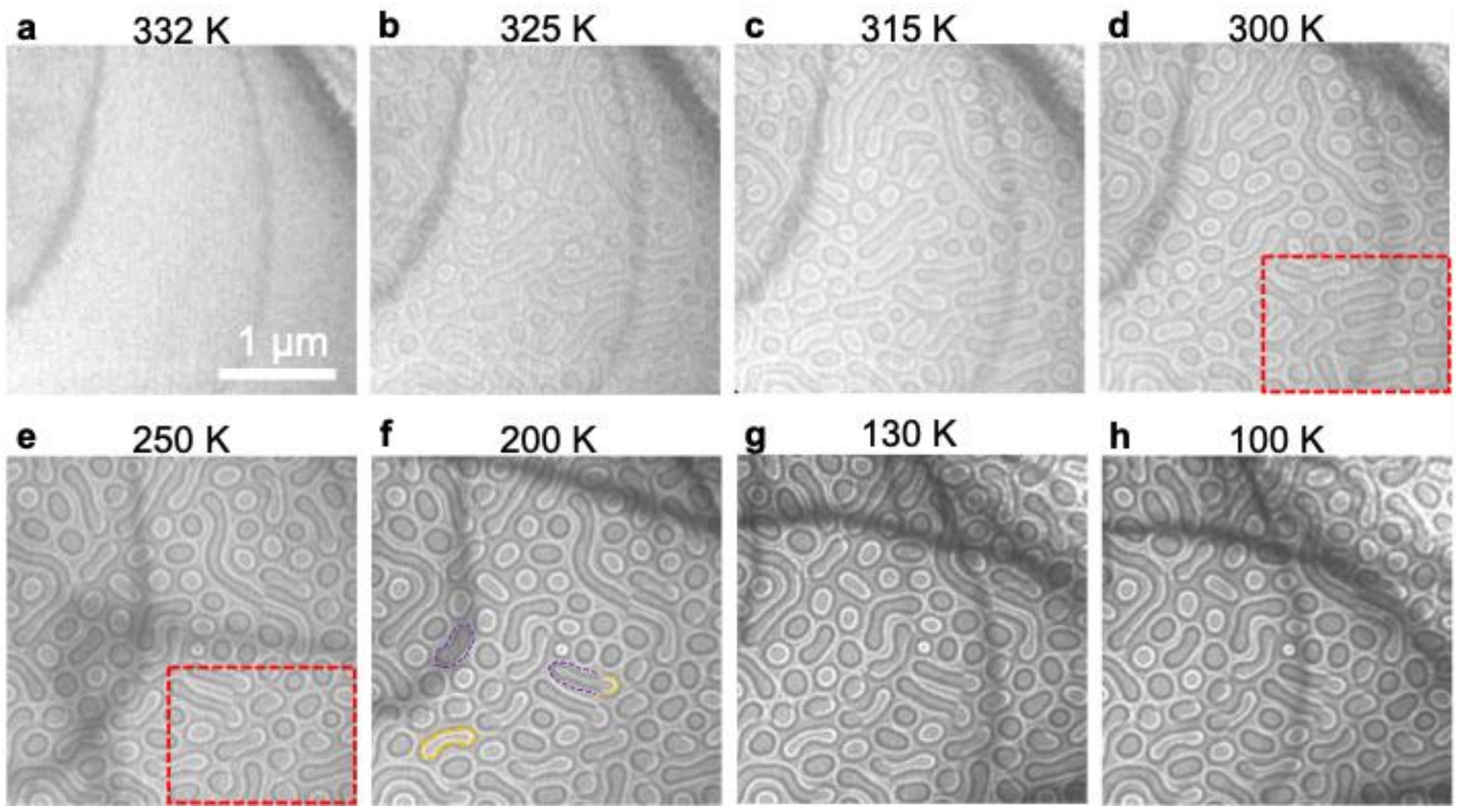

Fig. 2 | Formation of spontaneous skyrmions.

a Magnetic contrast is not observable when the sample is cooled to 332 K in the absence of a magnetic field. **b** Zero-field Lorentz TEM magnetic contrast of skyrmions, type-II bubbles and short spiral domains that appear at 325 K. **c-h** These contrasts are stable at 300 K, 250 K, 200 K, 130 K, 100 K, respectively. A slight change in magnetic contrast is observed at 250 K on the right corner of the LTEM image. In **f** the spin textures marked in orange or purple colours are elongated CW and CCW Bloch skyrmions, while an elongated topologically trivial bubble is marked by both orange and purple colours. The scale bar in **a** corresponds to 1 μm and is the same for all LTEM images. The black lines clearly visible in **a** are the bending contours and are present in all the LTEM images. Note that the spin textures' contrast changes slightly in the area indicated by the red rectangles, most likely as a result of changes in magnetic anisotropy with temperature.

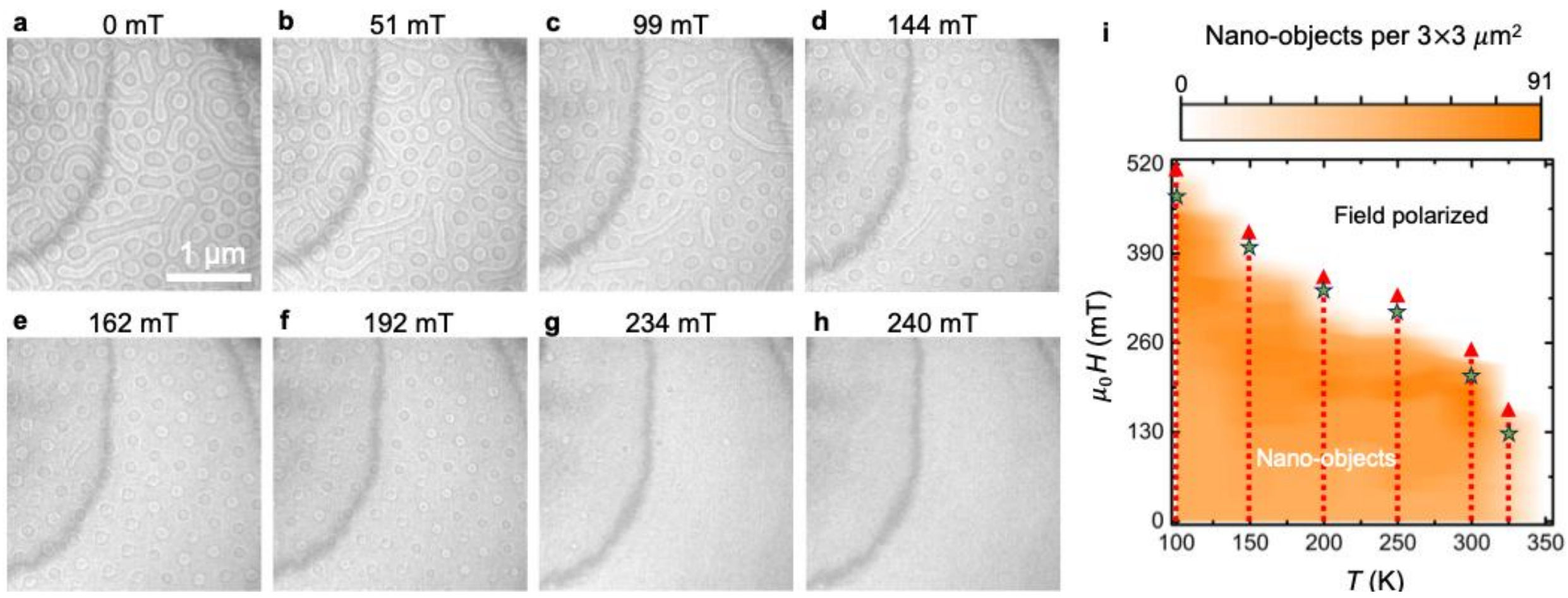

Fig. 3 | Field evolution of spontaneous spin textures at 300 K and magnetic phase diagram. **a** Zero-field spontaneous skyrmions at 300 K. **b-f** At higher fields, the spiral domains transform to circular nano-objects. **g, h** Sparse and field-polarized magnetic textures at 234 mT and 240 mT, respectively. The scale bar in **a** corresponds to 1 μm and is the same for all LTEM images. The black lines in each LTEM image correspond to bending contours. **i** Magnetic phase diagram that was determined by cooling the specimen from 365 K to various temperatures in the absence of a magnetic field. The number of nano-objects per 3 × 3 μm^2 area is indicated in the scale bar of **i**. The red dotted arrows in the phase diagram represents the field evolution of spontaneous skyrmions at different temperatures. FP corresponds to field polarization state. The magnetic field corresponding to the saturation magnetization at each investigated temperature to the phase diagram are indicated by green star symbols.

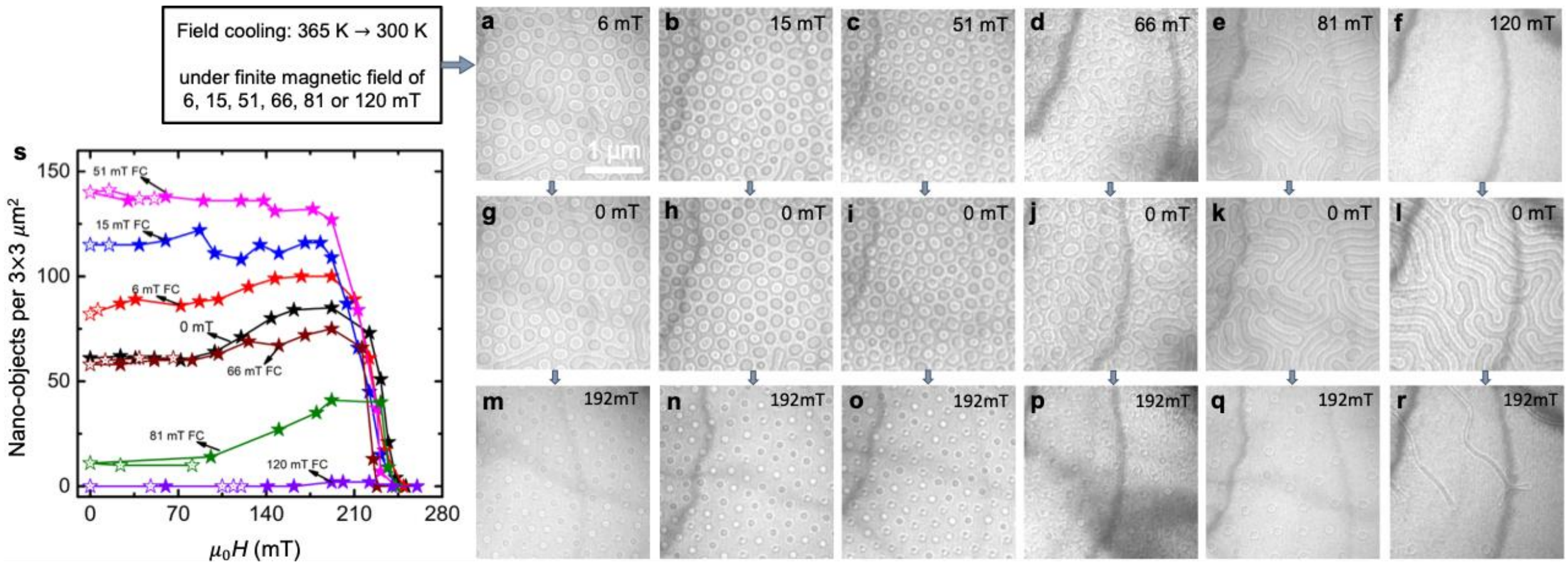

Fig. 4 | Stability after different field-cooling protocols.

The specimen is cooled down from 365 K to 300 K in the presence of various magnetic fields. **a-f** Upper panels: LTEM contrasts of the specimen after magnetic fields of 6 mT, 15 mT, 51 mT, 66 mT, 81 mT or 120 mT have been applied for field cooling, respectively. **g-l** Corresponding LTEM contrasts acquired after reducing the magnetic field to zero. **m-r** Corresponding LTEM contrasts taken after increasing the magnetic field to 192 mT. The scale bar in the upper panel of (a) corresponds to 1 μm and is the same for all LTEM images. Bending contours of black lines are clearly visible at each LTEM image. **s** Number of nano-objects found in the area of 3 μm × 3 μm for zero-field cooling (black-curve) and field cooling processes (values as indicated). The open stars correspond to fields for which field cooling was performed and then the field was reduced. The filled stars correspond to the case where the field was increased starting from zero field.

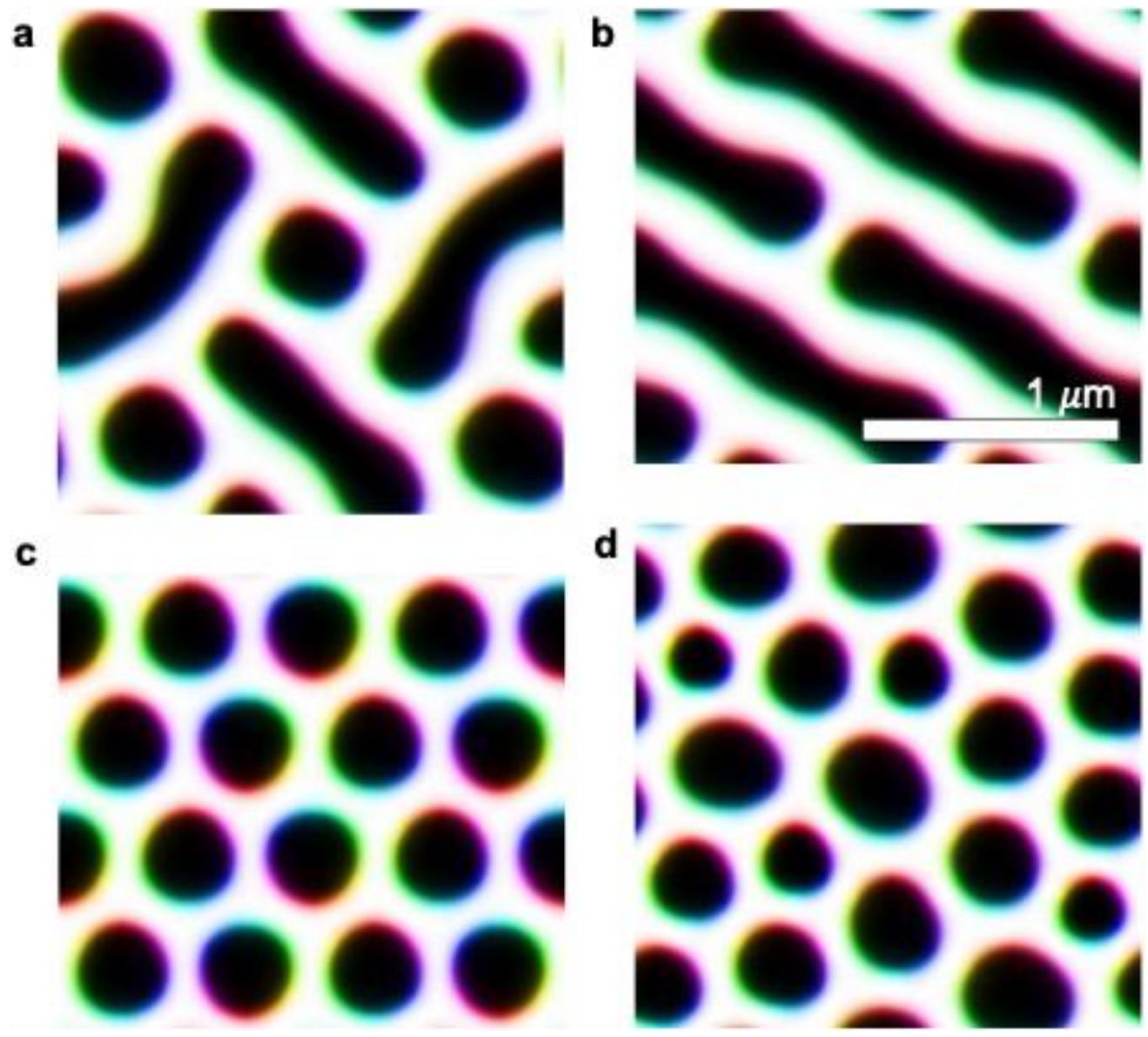

Fig. 5 | Various metastable spin textures in micromagnetic simulations.

a Starting from 9 Bloch skyrmions leads to a metastable configuration of 5 deformed skyrmions and 4 elongated skyrmions that form short stripe domains. **b** Four circular Bloch skyrmions elongate and form short stripe domains. **c** 16 Bloch skyrmions with helicities $\pm\pi/2$ form a periodic skyrmion lattice. **d** Starting from 36 Bloch skyrmions leads to a merging until only 20 Bloch skyrmions of various sizes form a metastable configuration. Periodic-boundary conditions have been used. For more details see Methods. The scale bar in **d** corresponds to 200 nm and is the same for all images.

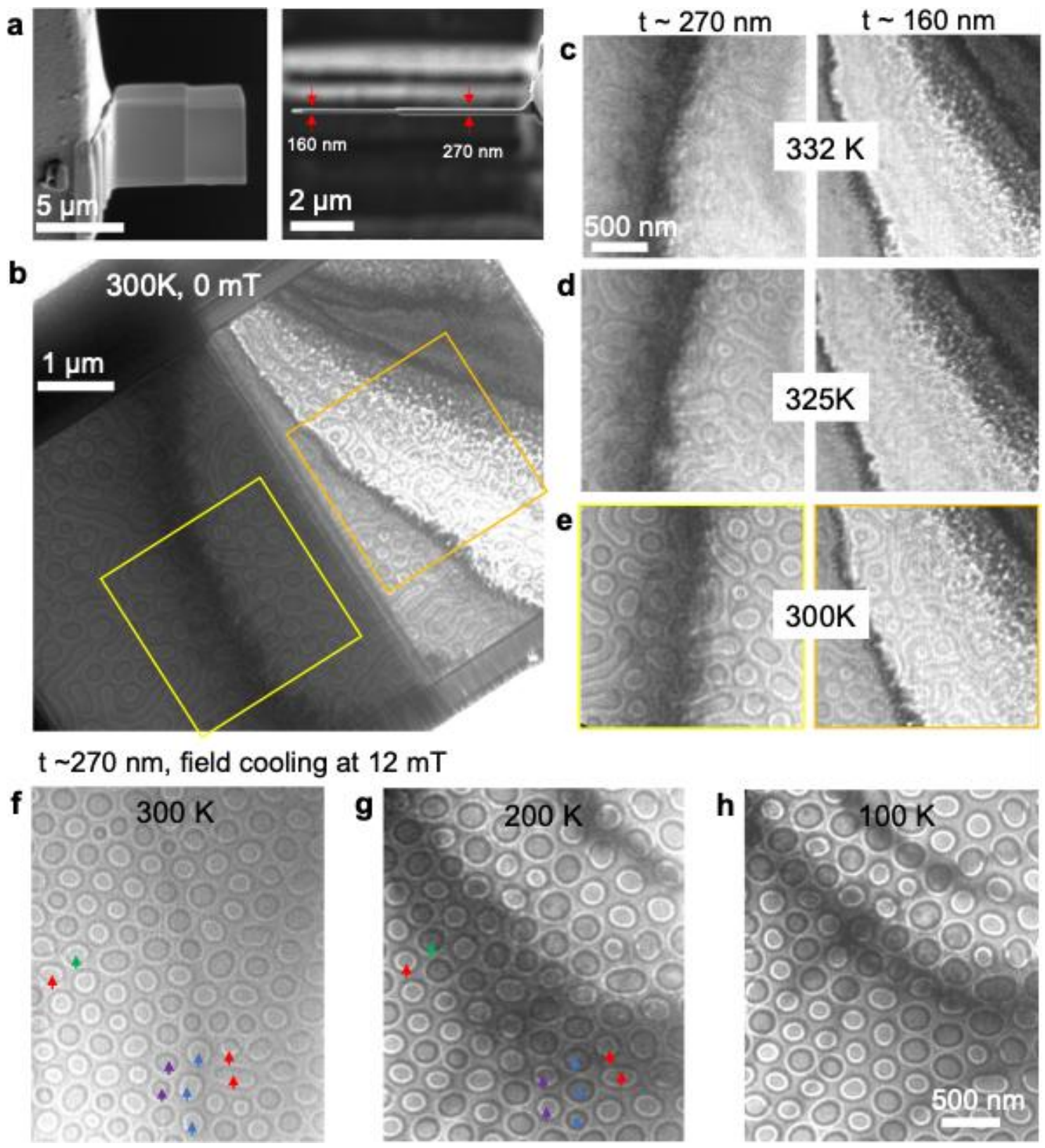

Fig. 6 | Stability of zero-field spin textures for thinner and thicker specimen.

a SEM images showing the side view (surface in the left image) and top view (different thicknesses in the right image) of the specimen. **b** The LTEM overview of the specimen at 300 K and 0 mT. Magnetic contrast is not visible in the upper corner of the specimen due to bending contours. The variation in image contrast originates from thickness variations within the TEM lamella. Reduced contrast in the thinner region results from a smaller integrated magnetic induction, whereas reduced contrast in the thicker region arises from decreased electron transmission. **c-e** LTEM contrasts captured at 332 K, 325 K and 300 K, respectively for thicker (270 nm, left side) and thinner (160 nm, right side) regions of the specimen. These images are taken from the same regions marked in yellow and orange. The scale bar for the left side LTEM image in **c** corresponds to 500 nm and is the same for the images in **c**-**e**. **f** LTEM contrast for the thicker region at 300 K after field cooling the specimen from 365 K at a constant magnetic field of 12 mT. **g, h** The specimen was consequently cooled to 200 K and 100 K, keeping the magnetic field constant. Differently colored arrows in **f**, **g** point to the same textures at different temperatures that have transformed (red: from trivial bubble to CW Bloch skyrmion, green: from trivial bubble to CCW Bloch skyrmion, blue: from CW Bloch skyrmion to CCW, purple: from CCW Bloch skyrmion to CW). The scale bar in **f** corresponds to 500 nm and is the same for the LTEM images in **g** and **h**.

References

[1] Parkin, S. S.; Hayashi, M.; Thomas, L. Magnetic domain-wall racetrack memory. *Science* **320**, 190-194, 2008.

[2] Fert, A.; Cros, V.; Sampaio, J. Skyrmions on the track. *Nat. Nanotechnol.* **8**, 152, 2013.

[3] Bogdanov, A.; Hubert, A. Thermodynamically stable magnetic vortex states in magnetic crystals. *J. Magn. Magn. Mater.* **138**, 255-269, 1994.

[4] MüHlbauer, S.; Binz, B.; Jonietz, F.; Pfleiderer, C.; Rosch, A.; Neubauer, A.; Georgii, R.; BöNi, P. Skyrmion lattice in a chiral magnet. *Science* **323**, 915-919, 2009.

[5] Dzyaloshinsky, I. A thermodynamic theory of "weak" ferromagnetism of antiferromagnetics. *J. Phys. Chem. Solids* **4**, 241-255, 1958.

[6] Moriya, T. Anisotropic superexchange interaction and weak ferromagnetism. *Phys. Rev.* **120**, 91, 1960.

[7] Yu, X.; Onose, Y.; Kanazawa, N.; Park, J.; Han, J.; Matsui, Y.; Nagaosa, N.; Tokura, Y. Real-space observation of a two-dimensional skyrmion crystal. *Nature* **465**, 901, 2010.

[8] Tokunaga, Y.; Yu, X.; White, J.; Rønnow, H. M.; Morikawa, D.; Taguchi, Y.; Tokura, Y. A new class of chiral materials hosting magnetic skyrmions beyond room temperature. *Nat. Commun.* **6**, 7638, 2015.

[9] Kézsmárki, I.; Bordács, S.; Milde, P.; Neuber, E.; Eng, L.; White, J.; Rønnow, H. M.; Dewhurst, C.; Mochizuki, M.; Yanai, K. Néel-type skyrmion lattice with confined orientation in the polar magnetic semiconductor GaV_4S_8. *Nat. Mater.* **14**, 1116-1122, 2015.

[10] Nayak, A. K.; Kumar, V.; Ma, T.; Werner, P.; Pippel, E.; Sahoo, R.; Damay, F.; Rößler, U. K.; Felser, C.; Parkin, S. S. Magnetic antiskyrmions above room temperature in tetragonal Heusler materials. *Nature* **548**, 561, 2017.

[11] Jena, J.; Stinshoff, R.; Saha, R.; Srivastava, A. K.; Ma, T.; Deniz, H.; Werner, P.; Felser, C.; Parkin, S. S. Observation of magnetic antiskyrmions in the low magnetization ferrimagnet $Mn_2Rh_{0.95}Ir_{0.05}Sn$. *Nano Lett.* **20**, 59-65, 2020.

[12] Karube, K.; Peng, L.; Masell, J.; Yu, X.; Kagawa, F.; Tokura, Y.; Taguchi, Y. Room-temperature antiskyrmions and sawtooth surface textures in a non-centrosymmetric magnet with S_4 symmetry. *Nat. Mater.* **20**, 335-340, 2021.

[13] Yu, X.; Mostovoy, M.; Tokunaga, Y.; Zhang, W.; Kimoto, K.; Matsui, Y.; Kaneko, Y.; Nagaosa, N.; Tokura, Y. Magnetic stripes and skyrmions with helicity reversals. *Proc. Natl. Acad. Sci.* **109**, 8856-8860, 2012.

[14] Phatak, C.; Heinonen, O.; De Graef, M.; Petford-Long, A. Nanoscale skyrmions in a nonchiral metallic multiferroic: Ni_2MnGa. *Nano Lett.* **16**, 4141-4148, 2016.

[15] Hou, Z.; Ren, W.; Ding, B.; Xu, G.; Wang, Y.; Yang, B.; Zhang, Q.; Zhang, Y.; Liu, E.; Xu, F. Observation of various and spontaneous magnetic skyrmionic bubbles at room temperature in a frustrated kagome magnet with uniaxial magnetic anisotropy. *Adv. Mater.* **29**, 1701144, 2017.

[16] Montoya, S.; Couture, S.; Chess, J.; Lee, J.; Kent, N.; Henze, D.; Sinha, S.; Im, M.-Y.; Kevan, S.; Fischer, P. Tailoring magnetic energies to form dipole skyrmions and skyrmion lattices. *Phys. Rev. B* **95**, 024415, 2017.

[17] Chakrabartty, D.; Jamaluddin, S.; Manna, S. K.; Nayak, A. K. Tunable room temperature magnetic skyrmions in centrosymmetric kagome magnet Mn_4Ga_2Sn. *Commun. Phys.* **5**, 189, 2022.

[18] Zheng, F.; Li, H.; Wang, S.; Song, D.; Jin, C.; Wei, W.; Kovács, A.; Zang, J.; Tian, M.; Zhang, Y. Direct imaging of a zero-field target skyrmion and its polarity switch in a chiral magnetic nanodisk. *Phy. Rev. Lett.* **119**, 197205, 2017.

[19] Peng, L.; Iakoubovskii, K. V.; Karube, K.; Taguchi, Y.; Tokura, Y.; Yu, X. Formation and Control of Zero-Field Antiskyrmions in Confining Geometries. *Adv. Sci.* **9**, 2202950, 2022.

[20] Jena, J.; Göbel, B.; Ma, T.; Kumar, V.; Saha, R.; Mertig, I.; Felser, C.; Parkin, S. S. Elliptical Bloch skyrmion chiral twins in an antiskyrmion system. *Nat. Commun.* **11**, 1115, 2020.

[21] Milde, P.; Köhler, D.; Seidel, J.; Eng, L.; Bauer, A.; Chacon, A.; Kindervater, J.; Mühlbauer, S.; Pfleiderer, C.; Buhrandt, S. Unwinding of a skyrmion lattice by magnetic monopoles. *Science* **340**, 1076-1080, 2013.

[22] Peng, L.; Zhang, Y.; Ke, L.; Kim, T.-H.; Zheng, Q.; Yan, J.; Zhang, X.-G.; Gao, Y.; Wang, S.; Cai, J. Relaxation dynamics of zero-field skyrmions over a wide temperature range. *Nano Lett.* **18**, 7777-7783, 2018.

[23] Saha, R.; Meyerheim, H. L.; Göbel, B.; Hazra, B. K.; Deniz, H.; Mohseni, K.; Antonov, V.; Ernst, A.; Knyazev, D.; Bedoya-Pinto, A. Observation of Néel-type skyrmions in acentric self-intercalated $Cr_{1+\delta}Te_2$. *Nat. Commun.* **13**, 3965, 2022.

[24] Birch, M.; Powalla, L.; Wintz, S.; Hovorka, O.; Litzius, K.; Loudon, J.; Turnbull, L.; Nehruji, V.; Son, K.; Bubeck, C. History-dependent domain and skyrmion formation in 2D van der Waals magnet Fe_3GeTe_2. *Nat. Commun.* **13**, 3035, 2022.

[25] Karube, K.; White, J. S.; Morikawa, D.; Bartkowiak, M.; Kikkawa, A.; Tokunaga, Y.; Arima, T.-H.; Rønnow, H. M.; Tokura, Y.; Taguchi, Y. Skyrmion formation in a bulk chiral magnet at zero magnetic field and above room temperature. *Phy. Rev. Mater.* **1**, 074405, 2017.

[26] Roessler, U. K.; Bogdanov, A.; Pfleiderer, C. Spontaneous skyrmion ground states in magnetic metals. *Nature* **442**, 797-801, 2006.

[27] Ozawa, R.; Hayami, S.; Motome, Y. Zero-field skyrmions with a high topological number in itinerant magnets. *Phys. Rev. Lett.* **118**, 147205, 2017.

[28] Heinze, S.; Von Bergmann, K.; Menzel, M.; Brede, J.; Kubetzka, A.; Wiesendanger, R.; Bihlmayer, G.; Blügel, S. Spontaneous atomic-scale magnetic skyrmion lattice in two dimensions. *Nat. Phys.* **7**, 713-718, 2011.

[29] Meyer, S.; Perini, M.; Von Malottki, S.; Kubetzka, A.; Wiesendanger, R.; Von Bergmann, K.; Heinze, S. Isolated zero field sub-10 nm skyrmions in ultrathin Co films. *Nat. Commun.* **10**, 3823, 2019.

[30] Zuo, S.; Liu, J.; Qiao, K.; Zhang, Y.; Chen, J.; Su, N.; Liu, Y.; Cao, J.; Zhao, T.; Wang, J. Spontaneous Topological Magnetic Transitions in $NdCo_5$ Rare-Earth Magnets. *Adv. Mater.* **33**, 2103751, 2021.

[31] Zuo, S.; Qiao, K.; Zhang, Y.; Zhao, T.; Jiang, C.; Shen, B. Spontaneous Biskyrmion Lattice in a Centrosymmetric Rhombohedral Rare-Earth Magnet with Easy-Plane Anisotropy. *Nano Lett.*, 2023.

[32] Zhao, X.; Tang, J.; Pei, K.; Wang, W.; Lin, S.-Z.; Du, H.; Tian, M.; Che, R. Current-induced magnetic skyrmions with controllable polarities in the helical phase. *Nano Lett.* **22**, 8793-8800, 2022.

[33] Song, Y.; Xu, T.; Zhao, G.; Xu, Y.; Zhong, Z.; Zheng, X.; Shi, N.; Zhou, C.; Hao, Y.; Huang, Q. High-density, spontaneous magnetic biskyrmions induced by negative thermal expansion in ferrimagnets. *Sci. Adv.* **9**, eadi1984, 2023.

[34] Rana, K. G.; Finco, A.; Fabre, F.; Chouaieb, S.; Haykal, A.; Buda-Prejbeanu, L. D.; Fruchart, O.; Le Denmat, S.; David, P.; Belmeguenai, M. Room-temperature skyrmions at zero field in exchange-biased ultrathin films. *Phy. Rev. Appl.* **13**, 044079, 2020.

[35] He, Y.; Fecher, G. H.; Kroder, J.; Borrmann, H.; Wang, X.; Zhang, L.; Kuo, C.-Y.; Liu, C.-E.; Chen, C.-T.; Chen, K. Easy-cone magnetic structure in $(Cr_{0.9}B_{0.1})$ Te. *Appl. Phys. Lett.* **116**, 102404, 2020.

[36] Pollard, S. D.; Garlow, J. A.; Yu, J.; Wang, Z.; Zhu, Y.; Yang, H. Observation of stable Néel skyrmions in cobalt/palladium multilayers with Lorentz transmission electron microscopy. *Nat. Commun.* **8**, 14761, 2017.

[37] Yu, X.; Koshibae, W.; Tokunaga, Y.; Shibata, K.; Taguchi, Y.; Nagaosa, N.; Tokura, Y. Transformation between meron and skyrmion topological spin textures in a chiral magnet. *Nature* **564**, 95-98, 2018.

[38] Tang, J.; Wu, Y.; Wang, W.; Kong, L.; Lv, B.; Wei, W.; Zang, J.; Tian, M.; Du, H. Magnetic skyrmion bundles and their current-driven dynamics. *Nat. Nanotechnol.* **16**, 1086-1091, 2021.

[39] Yu, X. Magnetic imaging of various topological spin textures and their dynamics. *J. Magn. Magn. Mater.* **539**, 168332, 2021.

[40] Tang, J.; Kong, L.; Wang, W.; Du, H.; Tian, M. Lorentz transmission electron microscopy for magnetic skyrmions imaging. *Chin. Phys. B* **28**, 087503, 2019.

[41] Jena, J.; Koraltan, S.; Bruckner, F.; Holst, K.; Tangi, M.; Claas, A.; Felser, C.; Suess, D.; Parkin, S. P. P. Topological phase transformation and collapse dynamics of spin textures in a non-centrosymmetric D_{2d} system. *Adv. Funct. Mater.,* **34**, 2403358, 2024.

[42] Jena, J.; Göbel, B.; Hirosawa, T.; Díaz, S. A.; Wolf, D.; Hinokihara, T.; Kumar, V.; Mertig, I.; Felser, C.; Lubk, A. Observation of fractional spin textures in a Heusler material. *Nat. Commun.* **13**, 2348, 2022.

[43] Wolf, D.; Schneider, S.; Rößler, U. K.; Kovács, A.; Schmidt, M.; Dunin-Borkowski, R. E.; Büchner, B.; Rellinghaus, B.; Lubk, A. Unveiling the three-dimensional magnetic texture of skyrmion tubes. *Nat. Nanotechnol.* **17**, 250-255, 2022.

[44] Liu, J.; Ding, B.; Liang, J.; Li, X.; Yao, Y.; Wang, W. Magnetic Skyrmionic bubbles at room temperature and sign reversal of the topological Hall effect in a layered ferromagnet $Cr_{0.87}Te$. *ACS Nano* **16**, 13911-13918, 2022.
